# Identification of the C9-hydrogenase for 9,17-dioxo-1,2,3,4,10,19-hexanorandrostan-5-oic acid (9,17-DOHNA) and the 7α-dehydratase essential for initiating β-oxidation of the B-, C-, and D-rings in steroid degradation by *Comamonas testosteroni* TA441

**DOI:** 10.1128/aem.02331-25

**Published:** 2026-04-03

**Authors:** Masae Horinouchi

**Affiliations:** 1 Surface and Interface Science Laboratory, RIKEN 13593, Wako, Saitama, Japan; Shanghai Jiao Tong University, Shanghai, China

**Keywords:** *Comamonas testosteroni*, bile acid, cholic acid, testosterone, cholesterol, steroid degradation, *Mycobacterium tuberculosis*, brain–gut–microbiome axis, HIP

## Abstract

*Comamonas testosteroni* TA441 is a model aerobic steroid-degrading bacterium whose sterane degradation pathway has been elucidated in the greatest detail to date. Similar pathways have been identified in many genera of bacteria, including both proteobacteria and actinobacteria, such as *Mycobacterium tuberculosis*. However, the genes encoding the C9-hydrogenase for 9,17-dioxo-1,2,3,4,10,19-hexanorandrostan-5-oic acid (9,17-DOHNA, also known as HIP) and the 7α-dehydratase essential for initiating β-oxidation of the B-, C-, and D-rings had not been identified. In this study, we identified these missing genes, located adjacent to the *chsE1E2H1H2ltp2* cluster involved in C17 side-chain degradation, and designated them *scdB* and *scdH*, respectively. This finding completes the elucidation of all degradation steps of 9,17-DOHNA prior to D-ring cleavage. AlphaFold models showed that ScdB and at least five hydrogenases/dehydrogenases involved in steroid degradation in TA441 share a similar dimer structures with Rossmann fold motif. In contrast, ScdH was predicted to form a homohexameric structure similar to ScdY and ScdN, involved in B-, C-, and D-ring degradation in TA441. Furthermore, AlphaFold modeling revealed that SteC, the dehydratase responsible for removing the C12β-hydroxyl group from 9,17-DOHNA derivatives, exhibits strong structural similarity to BaiE, the bile acid 7α-dehydratase of *Clostridium scindens* JCM 10418/VPI 12708, despite sharing only ~28% amino acid sequence identity.

**IMPORTANCE:**

Research on bacterial aerobic steroid degradation began more than 70 years ago, initially to produce intermediates for steroid drug synthesis. Recently, this field has gained renewed attention due to its implications for human health—for example, the role of cholesterol import and degradation in the persistence of *Mycobacterium tuberculosis* H37Rv within chronically infected lungs. *Comamonas testosteroni* TA441 serves as a key model organism for elucidating aerobic steroid degradation, with pathways for cleavage of the A-, B-, C-, and D-rings already well established. The functions and structures of the enzymes identified in TA441 display striking similarities to those in actinobacteria, such as *M. tuberculosis*. In this study, we identified two enzymes indispensable for initiating β-oxidation of the B-, C-, and D-rings, thereby filling the last remaining gaps for initiating this pathway. Our AlphaFold-based structural analysis of these enzymes not only provides new insights into the steroid metabolism of *M. tuberculosis* but also broadens understanding of the ecological and physiological significance of bacterial steroid degradation.

## INTRODUCTION

Steroid compounds perform a wide range of biological functions in plants and animals, including humans, where they serve essential roles as hormones, cholesterol, and bile acids (1–4). Aerobic degradation of steroids by bacteria has been recognized for over 70 years, with pioneering studies in the 1950s and 1960s using the two representative steroid-degrading bacteria, the actinomycete *Rhodococcus equi* and the pseudomonad *Comamonas testosteroni* (5–8).

**Fig 1:**
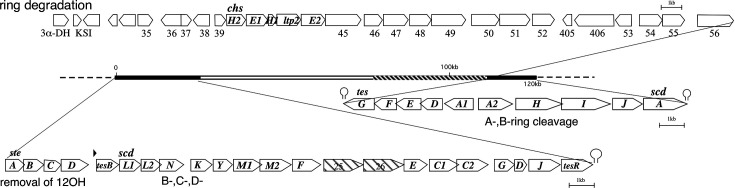
The 120-kb mega-cluster of steroid degradation genes in *C. testosteroni* TA441. The *tesG–scdA* cluster contains genes mainly responsible for aromatization of the A-ring (*tesH, tesI, tesJ*) and for ring cleavage and degradation of the A- and B-rings (*tesG, tesF, tesE, tesD, tesA1A2*). The *steA–tesR* cluster contains genes required for removal of the C12β hydroxyl group (*steABCD*) and genes involved in concurrent cleavage and β-oxidation of the C- and D-rings (*scdL1L2, scdN, scdK, scdY, scdM1M2, scdF, scdE, scdC1C2, scdG, scdD, scdJ*). *tesB* encodes the meta-cleavage enzyme for the aromatized A-ring, and *tesR* encodes the positive regulator of steroid degradation genes. *scdA* encodes the CoA-transferase initiating β-oxidation of the B-, C-, and D-rings. Other genes: 3α-DH, encoding 3α-hydroxydehydrogenase; KSI, 3-ketosteroid Δ4–5 isomerase; *chsH2, chsE1, chsH1, trp2,* and *chsE2,* which encode enzymes for removal of the isopropyl residue at C17. Genes between ORF35–55 are predicted to be steroid-degradation genes yet to be characterized.

Our previous studies elucidated the almost complete degradation pathways of the sterane structure—A-, B-, C-, and D-rings—as well as several side chains such as hydroxyl substituents and the C17 isopropyl residue in *C. testosteroni* TA441, establishing this strain as a model organism for aerobic bacterial steroid degradation (9–29). (The whole degradation pathway revealed in TA441 is shown in Fig. S1). Genes for A- and B-ring cleavage via A-ring aromatization and those for B-, C-, and D-ring degradation primarily via β-oxidation are located at both ends of the 120-kb “mega-cluster” of steroid degradation genes (Fig. 1).

In bacterial cholic acid degradation, the C17 side chain must be shortened to a ketone via a propionyl residue prior to sterane-ring degradation. Our most recent work demonstrated that this conversion is mediated by the dehydrogenase ChsE1E2, hydratase ChsH1H2, and lipid transfer protein Ltp2, which share high structural similarity with the corresponding enzymes in *Mycobacterium tuberculosis* (28–32). Aerobic steroid degradation pathways similar to that of TA441 are thought to exist in other aerobic steroid-degrading bacteria, including actinomycetes such as *Rhodococcus*, *Mycobacterium*, and *Sphingomonas* (33–36), as well as pseudomonadota such as *Pseudomonas* spp. (37), *Steroidobacter denitrificans* (38), *Caenibius tardaugens* (39), and other Comamonadaceae. In *M. tuberculosis* H37Rv, whose cholesterol import system is essential for persistence in chronically infected animal lungs (40), cholesterol catabolism is also critical for pathogenic maintenance in the host (41). Among at least 27 recognized *Comamonas* species, only *C. aquatica*, *C. kerstersii*, *C. terrigena*, and *C. testosteroni* are considered opportunistic pathogens, and only *C. thiooxydans* (42) and *C. resistens* (43, 44) have been reported to possess steroid degradation genes similar to those of *C. testosteroni*.

**Fig 2:**
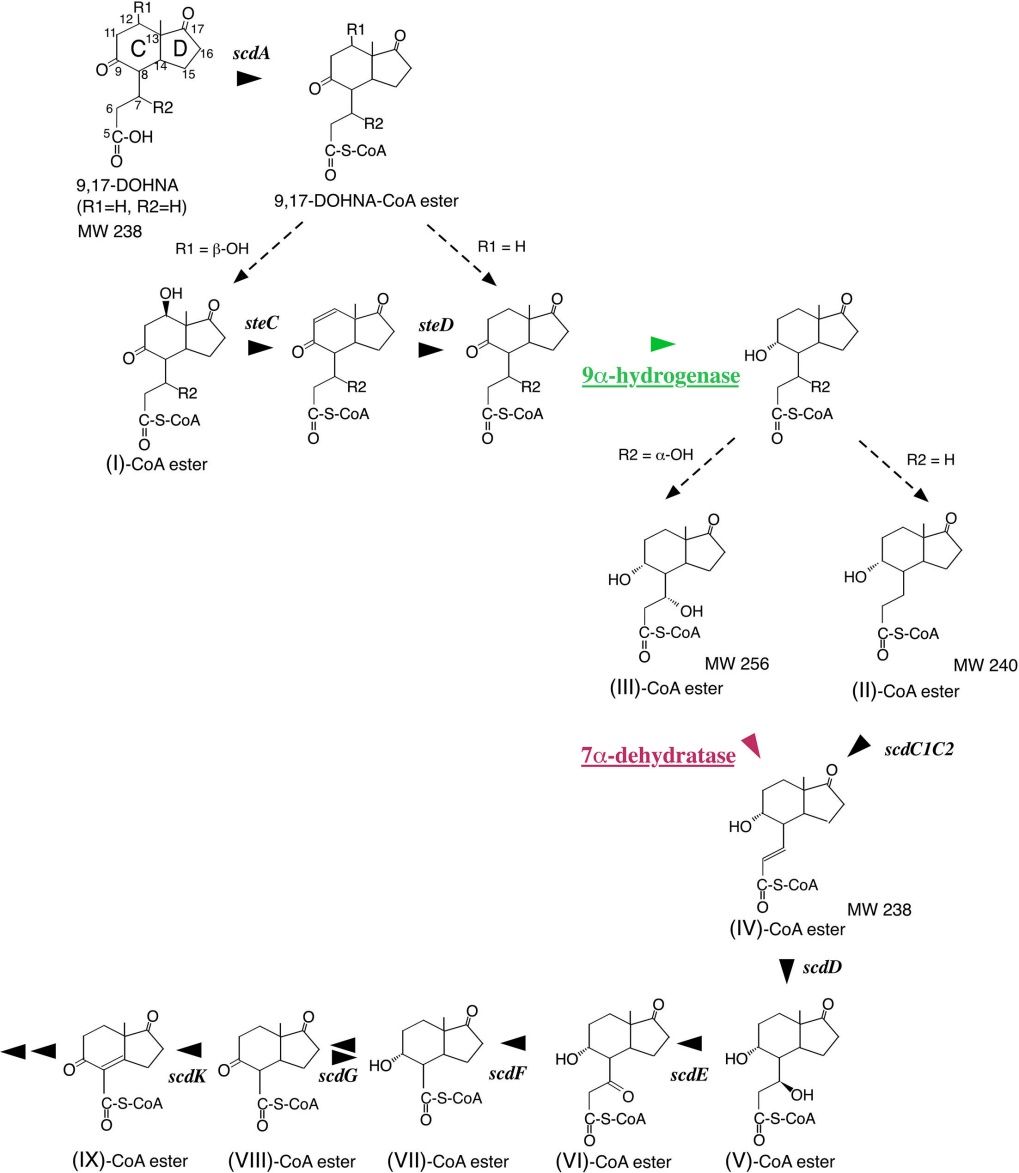
Steroid degradation pathway of the B-, C-, and D-rings from 9,17-dioxo-1,2,3,4,10,19-hexanorandrostan-5-oic acid (9,17-DOHNA) to **IX**-CoA ester, immediately before D-ring cleavage, in *C. testosteroni* TA441. Enzymes responsible for C9α-hydrogenase and C7α-dehydratase activities (indicated with color) remain unidentified. Compounds are 9,17-DOHNA; 9,17-dioxo-1,2,3,4,10,19-hexanorandrostan-5-oic acid (also known as 3aα-*H*-4α [3′-propionic acid]−7aβ-methylhexahydro-1,5-indanedione, HIP) (R1, R2=H), (**I**); 12β-hydroxy-9,17-dioxo-1,2,3,4,10,19-hexanorandrostan-5-oic acid (R2=H) , (**II**); 9α-hydroxy-17-oxo-1,2,3,4,10,19-hexanorandrostan-5-oic acid, (**III**); 7α,9α-dihydroxy-17-oxo-1,2,3,4,10,19-hexanorandrostan-5-oic acid, (**IV**); 9α-hydroxy-17-oxo-1,2,3,4,10,19-hexanorandrost-6-en-5-oic acid, (**V**); 7β,9α-dihydroxy-17-oxo-1,2,3,4,10,19-hexanorandrostan-5-oic acid, (**VI**); 9α-hydroxy-7,17-dioxo-1,2,3,4,10,19- hexanorandrostan-5-oic acid, (**VII**); 9α-hydroxy-17-oxo-1,2,3,4,5,6,10,19-octanorandrostan-7-oic acid, (**VIII**); 9,17-dioxo-1,2,3,4,5,6,10,19-octanorandrostan-7-oic acid, and (**IX**); 9,17-dioxo-1,2,3,4,5,6,10,19-octanorandrost-8(14)-en-7-oic acid. Enzymes are SteC (dehydratase for 12β-OH to produce a double at C10 [12]), SteD (reductase for a double at C10 [12] to a single bond), ScdA (CoA-transferase for 9,17-DOHNA), ScdC1C2 (Δ6-dehydrogenase for **II-**CoA ester), ScdD (**IV-**CoA ester Δ6-hydratase), ScdE (**V-**CoA ester dehydrogenase at C7), ScdF (**VI-**CoA ester thiolase/CoA-transferase), ScdG (hydrogenase primarily for 9-OH of **VII-**CoA ester), and ScdK (Δ8(14)-dehydrogenase for **VIII-**CoA ester).

During A-ring cleavage, aromatization of the A-ring and opening of the B-ring generate intermediates that undergo degradation analogous to the bacterial biphenyl degradation pathway, forming 9,17-dioxo-1,2,3,4,10,19-hexanorandrostan-5-oic acid (9,17-DOHNA; also known as HIP, 3aα-H-4α[3′-propionic acid]−7aβ-methylhexahydro-1,5-indanedione) and 2-hydroxyhexa-2,4-dienoic acid (Fig. 2; Fig. S1) (9–15, 17–19) The intermediate 9,17-DOHNA, containing the intact C- and D-rings and an opened B-ring, is converted into its CoA ester by ScdA, encoded in the A/B-ring cleavage cluster, and subsequently degraded by successive β-oxidation cycles mediated by Scd genes located in the B-, C-, and D-ring degradation cluster (Fig. 1) (20–27, 29).

Although the overall steroid degradation pathway in TA441 is well characterized, several expected enzymatic steps have remained unidentified. Here, we report the discovery of the C9-hydrogenase acting on 9,17-DOHNA-CoA and the 7α-dehydratase essential for initiating β-oxidation of the B,C,D-rings in steroid degradation by *C. testosteroni* TA441. We also present AlphaFold-based analyses of these enzymes together with previously characterized steroid-degrading enzymes of TA441.

## RESULTS

### Analysis of the culture of ORF38- and ORF39-disrupted mutants incubated with cholic acid and its analogs

*Comamonas testosteroni* TA441 degrades steroids through C17-side chain degradation (28), A,B-ring cleavage, which is analogous to the bacterial “meta-cleavage pathway” involved in the degradation of aromatic compounds such as biphenyl (9–14, 17, 18), followed by B-, C-, D-ring degradation via several cycles of β-oxidation (19, 21, 23–27, 29). The addition of CoA by ScdA to the C, D-ring–containing intermediate with a cleaved B-ring, 9,17-dioxo-1,2,3,4,10,19-hexanorandrostan-5-oic acid (9,17-DOHNA), also known by its IUPAC name 3aα-H-4α[3′-propionic acid]−7aβ-methylhexahydro-1,5-indanedione (HIP), initiates the B,C,D-ring degradation (Fig. 2). This step is followed by 9α-hydrogenation, which produces 9α-hydroxy-17-oxo-1,2,3,4,10,19-hexanorandrostan-5-oic acid (**II**). Removal of the 12β-hydroxyl group by SteC and SteD occurs prior to the 9α-hydrogenation (Fig. 2), as mutants disrupted in *scdC* (SteC^–^) and *scdD* (SteD^–^) accumulate compounds containing a ketone group at C9 (27–29). The 9α-hydrogenase acting on 9,17-DOHNA-CoA ester has not been identified; although ScdG exhibits 9α-hydrogenation/dehydrogenation activity, it is not the primary enzyme responsible for this step because the ScdG-disrupted mutant retains normal 9α-hydrogenation activity toward 9,17-DOHNA-CoA ester. After the 9α-hydrogenation step, a double bond is introduced by ScdC1C2 when the initial steroid lacks a hydroxyl group at C7, whereas a C7α-dehydratase introduces the double bond when the steroid contains an α-oriented hydroxyl group at C7 (Fig. 2) (16). C7α-dehydration was suggested to be reversible, as 7α,9α-dihydroxy-17-oxo-1,2,3,4,10,19-hexanorandrostan-5-oic acid (**III**) was detected in the culture of the *scdD*-disrupted mutant (ScdD^–^) incubated with testosterone (Fig. S2). However, the enzyme responsible for C7α-dehydration had not been identified previously.

**Fig 3:**
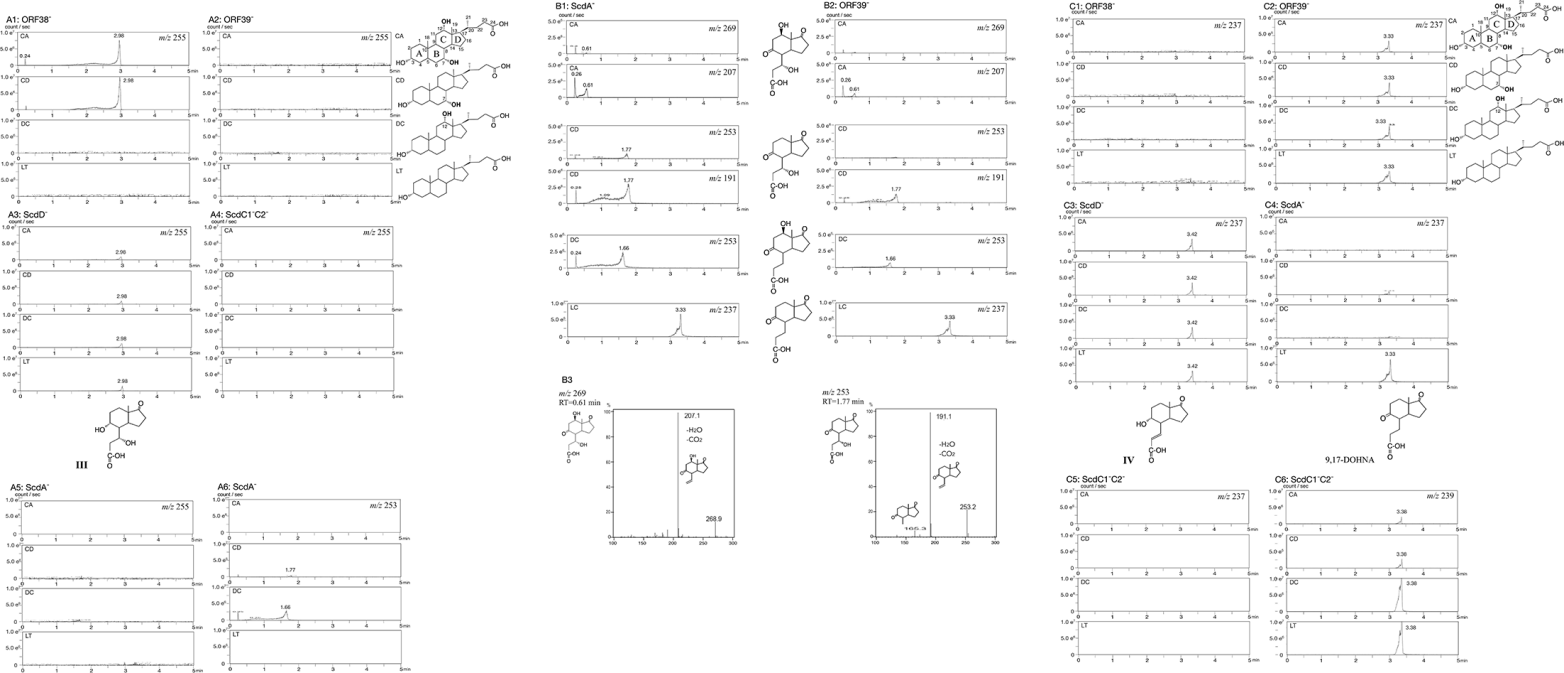
UPLC/MS analysis of ORF38^–^ and ORF39^–^ mutants incubated with 0.05% cholic acid (CA), chenodeoxycholic acid (CD), deoxycholic acid (DC), or lithocholic acid (LT) (the compound structural formulas are on the right side) for 7 days (**A1, A2, B1, B2**). Mutants ScdD^–^ (**A3**), ScdC1^–^C2^–^ (**A4**), and ScdA^–^ (**A5, A6**) were analyzed similarly for peak identification. Mutants were constructed by inserting a kanamycin resistance gene. Peaks in mass spectra represent: *m/z* 255 at RT = 2.98 min (7α,9α-dihydroxy-17-oxo-1,2,3,4,10,19-hexanorandrostan-5-oic acid, **III**) (A1CA, A1CD, and **A3**); *m/z* 253 at RT = 1.77 min (7α-hydroxy-9,17-dioxo-1,2,3,4,10,19-hexanorandrostan-5-oic acid) (A6 CD, B1CD, and B2 CD with mass spectrum in **B3**); *m/z* 253 at RT = 1.66 min (12β-hydroxy-9,17-dioxo-1,2,3,4,10,19-hexanorandrostan-5-oic acid) (A6 DC, B1DC, and B2 DC); *m/z* 269 at RT = 0.61 min (7α,12β-dihydroxy-9,17-dioxo-1,2,3,4,10,19-hexanorandrostan-5-oic acid) (B1CA and B2CA with mass spectrum in **B3**); *m/z* 237 at RT = 3.33 min (9,17-dioxo-1,2,3,4,10,19-hexanorandrostan-5-oic acid, 9,17-DOHNA) (B1LT, B2LT, **C2**, and C4LT); *m/z* 237 at RT = 3.42 min (9α-hydroxy-17-oxo-1,2,3,4,10,19-hexanorandrost-6-en-5-oic acid, **IV**) (**C3**); and *m/z* 239 at RT = 3.38 min (9α-hydroxy-17-oxo-1,2,3,4,10,19-hexanorandrostan-5-oic acid, **II**) (**C6**). All peaks labeled with retention times were not detected in cultures grown without steroid compounds. The vertical axis indicates intensity (counts/s), and the horizontal axis indicates retention time (min).

We analyzed the cultures of ORF38- and ORF39-disrupted mutants (ORF38^–^ and ORF39^–^, respectively) incubated with cholic acid as preliminary experiments. These ORFs are located just upstream of *chsH2E1H1ltp2chsE2*, which encode enzymes involved in degradation of the propionyl residue during C17-side chain breakdown (Fig. 1) (20, 23, 26, 28). The results suggested that ORF38 and ORF39 participate in the early steps of B,C,D-ring degradation. Homology searches indicated that ORF38 and ORF39 encode an enoyl-CoA hydratase and an SDR-family oxidoreductase, respectively. Therefore, we incubated ORF38^–^, ORF39^–^, and the mutants ScdA^–^, ScdC1C2^–^, and ScdD^–^ (Fig. 2) with cholic acid (CA), deoxycholic acid (DC), chenodeoxycholic acid (CD), and lithocholic acid (LT) for 7 days, followed by UPLC/MS analysis (Fig. 3, all the peaks labeled with RT were not detected in the culture without steroid compounds).

The ORF38^–^ mutant accumulated a large amount of a compound with *m/z* 255 at RT = 2.99 min in cultures supplemented with CA and CD, both possessing an α-oriented hydroxyl group at C7 but showed no notable accumulation of intermediate compounds with DC or LT, which lack a hydroxyl group at C7 (Fig. 3A. The MS/MS data are shown in Fig. S2). A small amount of **III** was detected in ScdD^–^ cultures, displaying the same RT (2.99 min) as that of the compound in the ORF38^–^ culture, suggesting that this compound corresponds to **III** and that the enzyme encoded by ORF38 is the dehydratase acting on the **III**-CoA ester. 9,17-DOHNA derivatives containing a 7α-hydroxyl and/or 12β-hydroxyl group (with *m/z* 269 or 253) and 9,17-DOHNA (*m/z* 237) were expected to be detected from the ORF38^–^ cultures incubated with CA, DC, CD, and LT, respectively. As shown in Fig. 3B, these compounds were indeed detected: the derivatives with 7α- and 12β-hydroxyl groups (*m/z* 269, RT = 0.61 min) and those with only the 7α-hydroxyl group (*m/z* 253, RT = 1.77 min) appeared predominantly as parent ions with *m/z* 207 and 191, respectively (Fig. 3B3) (27).

The ORF39^–^ mutant accumulated a compound with *m/z* 237 at RT = 3.33 min in all cultures containing cholic acid derivatives (Fig. 3C). Among the intermediate compounds shown in Fig. 2, both 9,17-DOHNA and 9α-hydroxy-17-oxo-1,2,3,4,10,19-hexanorandrost-6-en-5-oic acid (**IV**) have a molecular weight of 238. 9,17-DOHNA accumulates primarily in the ScdA^–^ mutant, while **IV** accumulates in the ScdD^–^ mutant, with the latter producing a distinct peak at *m/z* 175 (Fig. 3C. The MS/MS data are shown in Fig. S3). Therefore, the compound with *m/z* 237 at RT = 3.33 min in the ORF39^–^ culture is identified as 9,17-DOHNA, indicating that ORF39 encodes the 9α-hydrogenase responsible for the conversion of 9,17-DOHNA-CoA ester.

### Complementation experiments with ORF38 and ORF39 disrupted mutants

For further confirmation, the broad-host-range plasmid pMFYMhpRA (derived from pMFY42) (26, 45), serving as a negative control, and the same vector carrying either ORF38 (pMFYMhpORF38) or ORF39 (pMFYMhpORF39) were introduced into the ORF38^–^ and ORF39^–^ mutants, respectively (Table 1 and Table S1). pMFYMhpRA was designed to express cloned genes upon addition of 3-(3-hydroxyphenyl)propionic acid (3HPP), since in previous studies on sterane ring degradation, only partial activity recovery, sometimes hardly detectable recovery, was observed when the substrate and product of the target enzyme were CoA esters.

**TABLE 1:** Strains

Strain	Characteristic	Source or reference
TA441	Wild-type	(46, 47)
ORF38^-^	ORF38::Km^r^ mutant of TA441	This work
ORF39^-^	ORF39::Km^r^ mutant of TA441	This work
ScdA^-^	scdA::Km^r^ mutant of TA441	(15)
ScdC1^-^C2^-^	scdC1C2::Km^r^ mutant of TA441	(19)
ScdD^-^	scdD::Km^r^ mutant of TA441	(20, 22)

**Fig 4:**
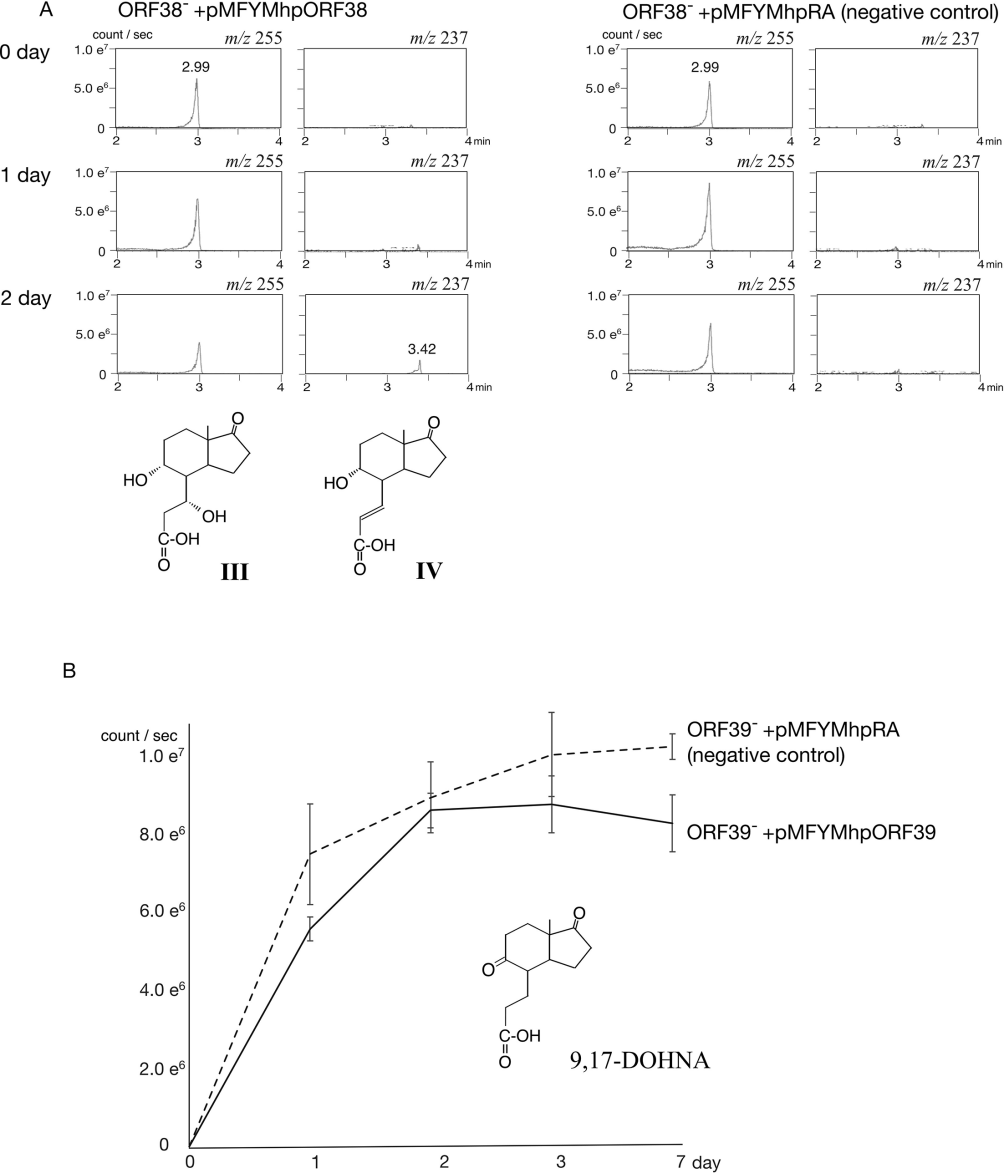
Complementation analysis of ORF38^–^ and ORF39^–^ mutants. (**A**) Mass chromatograms of ORF38^–^ carrying pMFYMhpORF38 and the vector control pMFYMhpRA, incubated with 0.05% LT for 0–2 days, showing peaks at *m/z* 255 (**III**) and *m/z* 237 (**IV**). (**B**) Amount of 9,17-DOHNA accumulated in ORF39^–^ expressing pMFYMhpORF39 or vector control, incubated with 0.05% 1,4-androstadien-3,17-dion (ADD) for 7 days. Quantification was based on mass chromatograms.

The ORF38^–^ and ORF39^–^ mutants were cultured with CD and LT, respectively, for 1 day before adding 3HPP, and the cultures were analyzed by UPLC/MS at appropriate time intervals. Figure 4A shows chromatograms of *m/z* 255 (**III**) and *m/z* 237 (**IV**) from cultures of ORF38^–^ carrying either pMFYMhpORF38 or the empty vector pMFYMhpRA. Compound **IV** was detected only in the complemented strain, demonstrating that the enzyme encoded by ORF38 catalyzes dehydration of **III**-CoA ester to **IV**-CoA ester. This enzyme was thus designated ScdH.

In contrast, chromatograms of ORF39^–^ carrying pMFYMhpORF39 and ORF39^–^ carrying the control vector showed only minor differences even after induction with 3HPP. To validate the catalytic activity, we tested the conversion of 9,17-DOHNA-CoA ester using crude extracts of *E. coli* harboring pUCORF39 (a pUC19-based plasmid carrying ORF39, Table S1). ORF39^–^ was cultured with 0.1% 1,4-androstadien-3,17-dione (ADD) for 7 days, then sonicated, and the supernatant was used as substrate. ADD was used because it is more readily converted to 9,17-DOHNA by TA441 than LT. *E. coli* strains carrying either pUCORF39 or pUC19 (control) were cultured with ampicillin and IPTG for 1 day, sonicated, and incubated with the substrate solution at 37°C for 1 h. HPLC/MS analysis showed that complementation with pUCORF39 resulted in an approximately 15% reduction in the amount of 9,17-DOHNA compared with the control strain (1.44 × 10⁷ ± 3.42 × 10⁶ vs. 1.75 × 10⁷ ± 3.53 × 10⁶ counts/s).

Because the decrease was modest, additional experiments were conducted with ORF39^–^ carrying pMFYMhpORF39 and the control strain, using ADD as substrate without tetracycline supplementation, since tetracycline was found to inhibit dehydrogenase ChsE1E2 in previous studies (28). Repeated experiments confirmed that the amount of 9,17-DOHNA was consistently lower in the complemented strain (Fig. 4B). Thus, ORF39 was identified as encoding the 9α-hydrogenase responsible for converting 9,17-DOHNA-CoA ester to **III-**CoA (C7=αOH) and/or **II**-CoA (C7=H), and this enzyme was named ScdB. During C- and D-ring degradation, ScdG functions as a 9α-dehydrogenase/hydrogenase, catalyzing the oxidation of 9α-hydroxy-17-oxo-1,2,3,4,5,6,10,19-octanorandrostan-7-oic acid (**VII**)-CoA ester to 9,17-dioxo-1,2,3,4,5,6,10,19-octanorandrostan-7-oic acid (**VIII**)-CoA ester, and vice versa. The conversion experiments yielded similarly small amounts of product (21), likely because CoA esters possessing a C9-ketone group and a C8–14 single bond are unstable and readily interconvert with compounds containing a C9-ketone and a C8–14 double bond or a C9-hydroxyl and a C8–14 single bond during steroidal C- and D-ring degradation in TA441 (21, 26). Like ScdG, ScdB is also highly likely a reversible enzyme, and in the case of ScdB, the product **III-**CoA (C7=αOH) and/or **II**-CoA (C7=H) will be readily converted to the following compounds, while CoA will be removed from 9,17-DOHNA-CoA ester when it accumulated excessively in the mutant culture, which is thought to be resulting in difficulty of detecting the conversion by ScdB.

### AlphaFold structures of ScdB and ScdH; comparison with other hydrogenases and hydratases in steroid degradation

**Fig 5:**
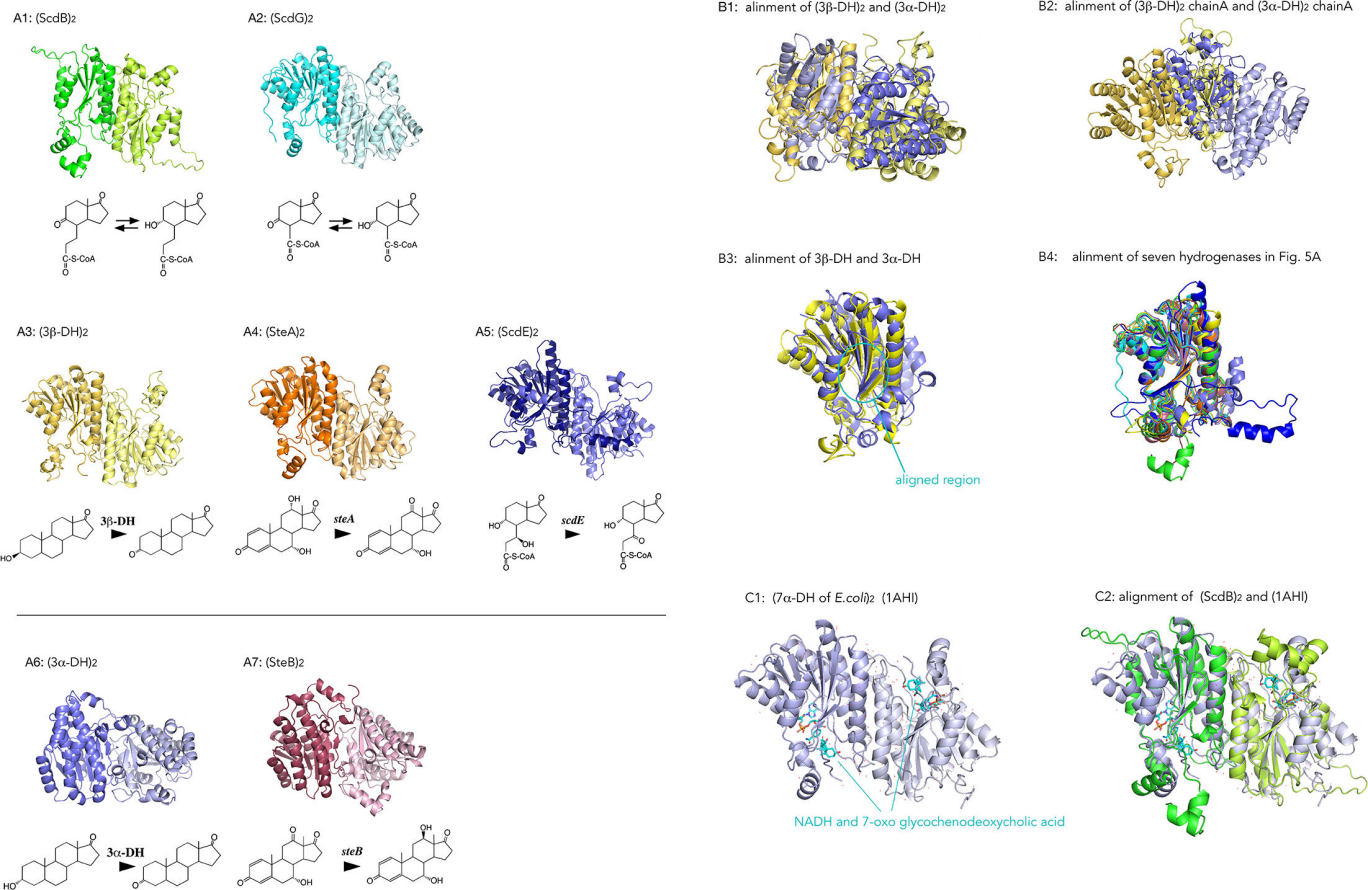
(**A**) AlphaFold models of (ScdB)₂ (**A1**) and other hydrogenases/dehydrogenases involved in interconversion of hydroxyl and ketone groups, (ScdG)_2_ (**VII**-CoA ester to **VI**-CoA ester) (**A2**), (ScdE)_2_ (**V**-CoA ester to **VI**-CoA ester) (**A3**), (3β-DH)_2_ (3β-dehydrogenase for compounds with sterane structure) (**A4**), (SteA)_2_ (12α-dehydrogenase) (**A5**), (3α-DH)_2_ (3α-dehydrogenase for compounds with sterane structure) (**A6**), and (SteB)_2_ (12β-hydrogenase) (**A7**). Reactions catalyzed by each enzyme are shown below the models. Expected position errors and alignments of top models are shown in Fig. S4A and C. (**B**) alignment of (3β-DH)₂ and (3α-DH)₂ (**B1**), alignment of one of the chains of (3β-DH)₂ and (3α-DH)₂ (**B2**), alignment of the amino acid residues 160–200 of the monomers (indicated with light blue circle) (**B3**), and monomer-level alignment of all enzymes in Fig. 5A (**B4**). (**C)** Crystal structure of 7α-hydroxysteroid dehydrogenase (7α-DH) from *E. coli* with NADH and the substrate steroid molecules (7-oxo glycochenodeoxycholic acid) (PDB: 1AHI) (49) (**C1**), and alignment of (ScdB)_2_ with the crystal structure of (7α-DH)_2_ (PDB: 1AHI) (RMSD = 0.85 Å over 227 Cα atoms) (**C2**).

ScdB identified in this study catalyzes the similar reaction, interconversion between a β-oriented hydroxyl group and a ketone group at C9, to previously identified ScdG (Fig. 1) (21). Homology search indicated that both ScdB and ScdG belong to the SDR family of oxidoreductases; therefore, we compared the AlphaFold (48) models of (ScdB)₂ and (ScdG)₂ (Fig. 5A1 and A2; expected position errors and alignments of the five top models are shown in Fig. S4A1 and A2, S4C1 and C2). Both ScdB and ScdG were predicted to function as homodimers, and their overall folds were similar, each exhibiting the characteristic Rossmann-like α/β/α sandwich fold typical of SDR oxidoreductases.

In contrast, the AlphaFold model of (ScdK)₂—an enzyme involved in the downstream reaction after ScdG (Fig. 1) and belonging to the NAD(P)H-dependent flavin oxidoreductase YrpB nitropropane dioxygenase family—was markedly different from those of ScdB and ScdG (Fig. S5A1; expected position errors and alignments of the top models in Fig. S5B). In *M. tuberculosis*, the enzymes corresponding to ScdG and ScdK were reported to function in the opposite order, catalyzing C8–14 dehydrogenation and 9α-hydrogenation/dehydrogenation, respectively (34). The AlphaFold model of only SteD (C11 alkene reductase; Fig. 1) resembled ScdK overall among hydrogenases/dehydrogenases for steroid degradation identified to date in TA441, comprising eight α-helices forming a ring-like structure (Fig. S5A2 and B), but alignment of ScdK and SteD was unsuccessful.

In addition to (ScdB)₂ and (ScdG)₂, we predicted the 3D structures of all hydrogenases/dehydrogenases identified to date in TA441 that catalyze interconversion between a ketone group and a hydroxyl group during steroid degradation. These included (3β-DH)₂ (3β-dehydrogenase for steranes), (SteA)₂ (12α-dehydrogenase for steranes), (3α-DH)₂ (3α-dehydrogenase for steranes), (SteB)₂ (12β-hydrogenase for steranes), and (ScdE)₂ (responsible for converting 7β,9α-dihydroxy-17-oxo-1,2,3,4,10,19-hexanorandrostan-5-oic acid (**V**)–CoA ester to 9α-hydroxy-7,17-dioxo-1,2,3,4,10,19-hexanorandrostan-5-oic acid (**VI**)–CoA ester) (Fig. 5A3 through A7; alignments of the five top models are shown in Fig. S4C) (14, 16, 17). All of these enzymes exhibited a Rossmann-like α/β/α sandwich fold characteristic of SDR-family oxidoreductases, and all were predicted to form dimers. The AlphaFold models of (3β-DH)₂, (SteA)₂, and (ScdE)₂ closely resembled those of (ScdB)₂ and (ScdG)₂, whereas the models of (3α-DH)₂ and (SteB)₂ showed different overall architectures and were more similar to each other (Fig. 5A6 and A7; see also alignments in Fig. S4B and C). Notably, models 0-2 of (SteB)₂ resembled all models of (3α-DH)₂, while models 3 and 4 of (SteB)₂ were more similar to (ScdB)₂ (Fig. S4C7).

When the dimers of (3α-DH)₂ and (3β-DH)₂ were aligned, the overall complexes appeared dissimilar (Fig. 5B1). However, one monomeric subunit aligned well between the two dimers (Fig. 5B2). Alignment at the monomer level suggested that 3α-DH and 3β-DH share highly similar three-dimensional structures; nevertheless, the RMSD was 10.0 Å over 181 Cα atoms. Therefore, we focused on the conserved putative active-site region located on the β-sheet (amino acid residues 158–198). Alignment of this region yielded an RMSD of 2.7 Å over 36 Cα atoms (Fig. 5B3). Extending this approach, we aligned all hydrogenases shown in Fig. 5A as monomers, demonstrating that they share a conserved three-dimensional architecture (Fig. 5B4). Superposition of ScdB with the other hydrogenases gave the following RMSD values (whole monomer alignment): ScdG (0.769 Å over 159 Cα atoms), ScdE (1.034 Å over 151 Cα atoms), 3β-DH (0.788 Å over 165 Cα atoms), SteA (0.716 Å over 177 Cα atoms), 3α-DH (1.556 Å over 138 Cα atoms), and SteB (0.710 Å over 169 Cα atoms).

In contrast to the marked difference between 3β-DH and 3α-DH at the dimer level, ScdB and 3α-DH exhibited similarity across the entire monomer structure. We subsequently aligned the amino acid sequences of these seven enzymes to identify specific regions responsible for structural divergence at the dimer level; however, no clear determinants were identified (Fig. S4D). Notably, 3α-DH lacks amino acids around residue 50 and contains an additional 28 amino acids around residues 127–154, which may contribute to the observed structural differences.

For further analysis, we compared the model of (ScdB)₂ with the crystal structure of 7α-hydroxysteroid dehydrogenase (7α-DH) from *Escherichia coli*. The 7α-DH catalyzes the dehydrogenation of a hydroxyl group at the 7α position of steroid substrates using NAD^+^ or NADP^+^ as a cofactor, and its crystal structure has been determined in complex with NADH and a steroid substrate (PDB: 1AHI) (49) (Fig. 5C1). Superposition of the (ScdB)₂ model with the crystal structure of (7α-DH)₂ gave an RMSD of 0.85 Å over 227 Cα atoms (Fig. 5C2). The alignment suggested that the substrate binds near the conserved β-sheet region of ScdB.

ScdH, ChsH1H2, ScdD (hydratase acting on the C6–7 double bond of IV-CoA ester), ScdY (hydratase acting on the C8–14 double bond of 17-dihydroxy-9-oxo-1,2,3,4,5,6,10,19-octanorandrost-8(14)-en-7-oic acid-CoA ester), ScdN (hydratase in the β-oxidation steps following C-ring cleavage), and SteC (dehydratase for the C12β-hydroxyl group) constitute the hydratases/dehydratases identified to date in steroid degradation in TA441 (Fig. 1 and 2, and Fig. S1) (29).

Homology analysis indicated that ScdH, ScdY, and ScdN belong to the crotonase-like enoyl-CoA hydratase/isomerase family, whereas ScdD and ChsH1H2 belong to the MaoC-family dehydratases. SteC was suggested to be a polyketide cyclase belonging to the SnoaL/NTF2 family.

**Fig 6:**
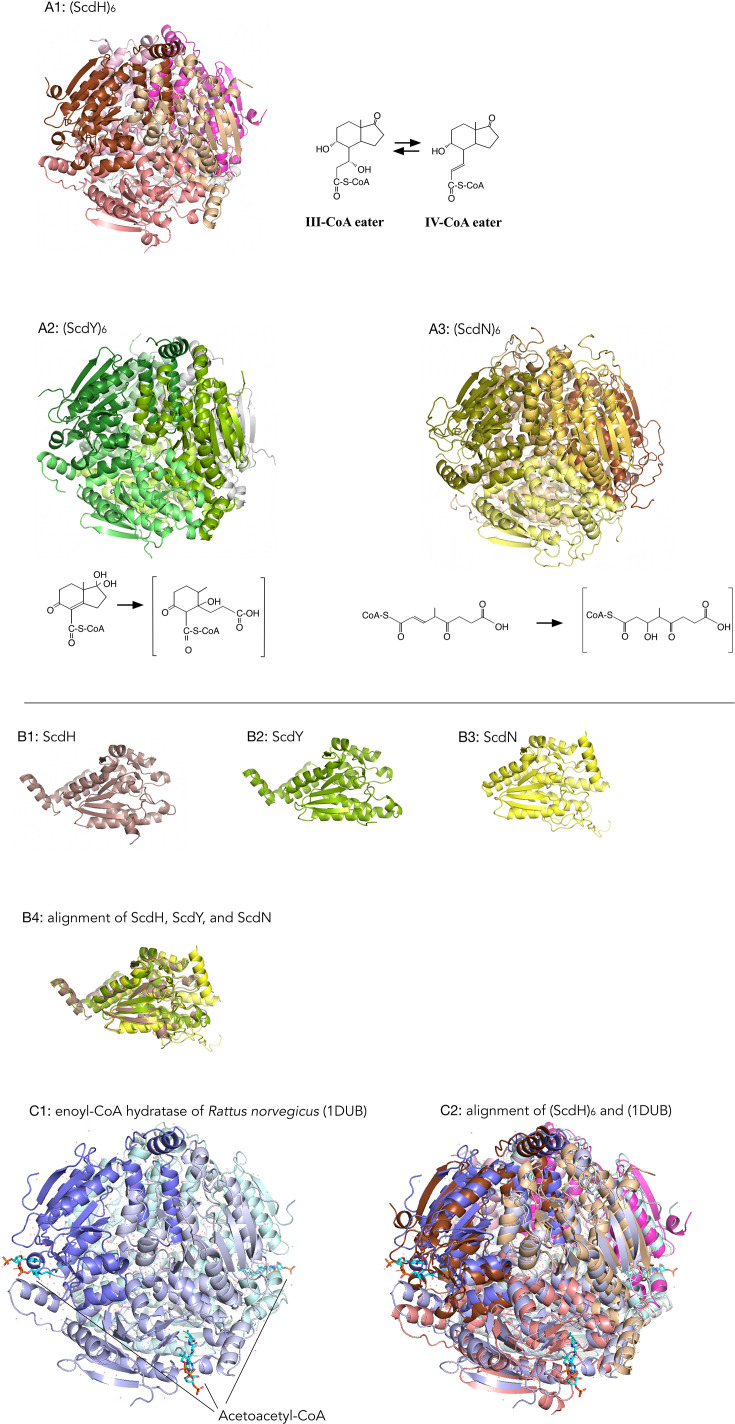
(**A**) AlphaFold models of (ScdH)_6_ (**A1**), (ScdY)_6_ (hydratase for the double bond at C8-14) (**A2**), and (ScdN)_6_ (hydratase for β-oxidation process after C-ring cleavage) (**A3**). (**B**) AlphaFold models of monomers ScdH (**B1**), ScdY (**B2**), ScdN (**B3**), and alignment of ScdH, ScdY, and ScdN monomers (**B4**). RMSD value of ScdH and ScdY gave 1.20 Å over 205 Cα atoms, and RMSD value of β-sheets of ScdH and ScdN gave 0.82 Å over 152 Cα atoms. Expected position errors and alignments of top models are shown in Fig. S6A and B. (**C**) Crystal structure of the enoyl-CoA hydratase from *Rattus norvegicus* with the substrate molecules (acetoacetyl-CoA) (PDB: 1DUB) (51) (**C1**), and alignment of (ScdH)_6_ with the crystal structure of the enoyl-CoA hydratase *R. norvegicus* (1DUB) (RMSD = 1.65 Å over 909 Cα atoms) (**C2**).

Crotonase-like hydratases typically form homohexamers (50), and AlphaFold similarly predicted hexameric assemblies for ScdH, ScdY, and ScdN (Fig. 6A1 through A3; expected position errors and alignments of the five top models are shown in Fig. S6A1 through A3 and S6B1 through B3, together with Clustal alignments in Fig. S6A5). Because hexameric structures are complex and difficult to compare directly (cf. alignment of (ScdH)₆ and (ScdY)₆ in Fig. S6A4), we aligned their monomers instead (Fig. 6B1 through B4). ScdH and ScdY were highly similar throughout (RMSD = 1.202 Å over 205 Cα atoms), whereas ScdN shared local similarity around the β-sheet region and the surrounding α-helix regions (RMSD = 0.82 Å over 152 Cα atoms) (Fig. 6B4). To speculate on the active site of (ScdH)₆, we searched the RCSB Protein Data Bank for hydratases with available crystal structures in complex with substrates and showing amino acid sequence homology. Unexpectedly, the closest match was enoyl-CoA hydratase from *Rattus norvegicus* (PDB: 1DUB) (51) (Fig. 6C1). Superposition of (ScdH)₆ with enoyl-CoA hydratase (1DUB) gave an RMSD of 1.650 Å over 909 Cα atoms (Fig. 6C2). Despite the large size of these enzymes, the RMSD was low, suggesting that the substrate-binding region of ScdH is structurally similar to that of the rat enzyme.

**Fig 7:**
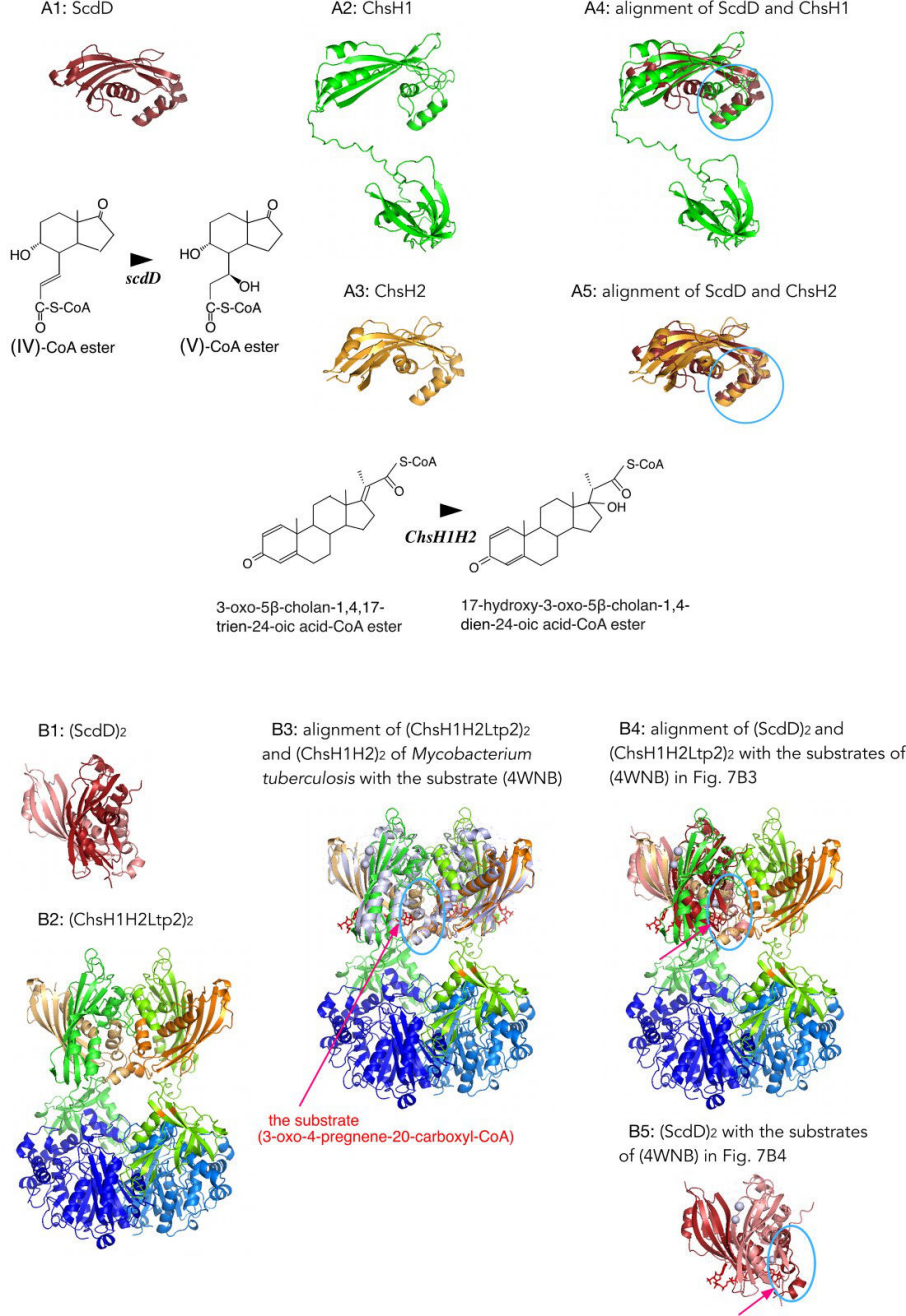
(**A**) AlphaFold models of ScdD (**A1**), ChsH1 (**A2**), ChsH2 (**A3**), alignment of initial around 60 amino acids of ScdD and ChsH1 (RMSD = 0.94 Å over 23 cα atoms) (**A4**), and alignment of initial around 60 amino acids of ScdD and ChsH2 (RMSD = 1.51 Å over 37 cα atoms) (**A5**). The aligned region is indicated with a light blue circle. Expected position errors and alignments of five top models are shown in Fig. S7A and B. (**B**) AlphaFold models of (ScdD)_2_ (**B1**), hetero hexamer (ChsH1H2Ltp2)_2_ (**B2**), alignment of (ChsH1H2Ltp2)_2_ and the (ChsH1H2)₂ of *M. tuberculosis* with the substrate (3-oxo-4-pregnene-20-carboxyl-CoA) (PDB: 4WNB) (52) (RMSD = 0.61 Å over 94 Cα atoms) (**B3**), alignment of amino acid residues 7–70 of (ScdD)_2_ and amino acid residues 1–63 of ChsH1 in the model in Fig. 7**B3** without (4wnb) (RMSD = 1.53 Å over 38 Cα atoms) (**B4**) and (ScdD)_2_ with the substrates in Fig. 7B4 (**B5**). The putative substrate binding region is indicated with light blue circles.

Alignments of ScdH with ScdD, ChsH1, and SteC showed no detectable similarity. In contrast, alignment of ScdD with ChsH1 and ChsH2 revealed structural resemblance among ScdD, the MaoC domain of ChsH1 (ChsH1_MaoC_), and ChsH2 (Fig. 7A1 through A3; expected position errors and alignments of the five top models of ScdD are shown in Fig. S7A and B1 and those of Chs enzymes were presented in the previous study (28)), although the RMSD values were high. Clustal alignments indicated that the N-terminal ~60 amino acids of ScdD were relatively similar to those of ChsH1 and ChsH2 (Fig. S7C1 and C2). Alignment of these regions showed that the initial ~60 residues of ScdD and ChsH1 monomers superposed with an RMSD of 0.94 Å over 23 Cα atoms, and those of ScdD and ChsH2 with an RMSD of 1.51 Å over 37 Cα atoms (Fig. 7A4 and A5; aligned regions indicated by a light blue circle). AlphaFold predicted ScdD to form a dimer (Fig. 7B1; Fig. S7A, B1 through B3), whereas previous studies showed that ChsH1H2 together with Ltp2 forms a heterohexamer (ChsH1H2Ltp2)₂, similar to the *Mycobacterium tuberculosis* enzyme (Fig. 7B2) (28). Superposition of (ChsH1H2)₂ from TA441 with the substrate-bound structure of (ChsH1H2)₂ from *M. tuberculosis* (PDB: 4WNB) (52) gave an RMSD of 0.61 Å over 94 Cα atoms (Fig. 7B3). This notably low RMSD indicates that substrate molecules of (ChsH1H2Ltp2)₂ in TA441 likely bind in positions similar to those in *M. tuberculosis*. Superimposition of the (ScdD)₂ model onto the hexamer model shown in Fig. 7B3 demonstrated structural similarity between ScdD and ChsH1_MaoC_ within the hexameric assembly (Fig. 7B4). Although no crystal structure of a substrate-bound hydratase homologous to ScdD was identified in the RCSB search, the (ScdD)₂ model with substrate (Fig. 7B4) suggested that the region aligned in Fig. 7A corresponds to the substrate-binding region (Fig. 7B5).

**Fig 8:**
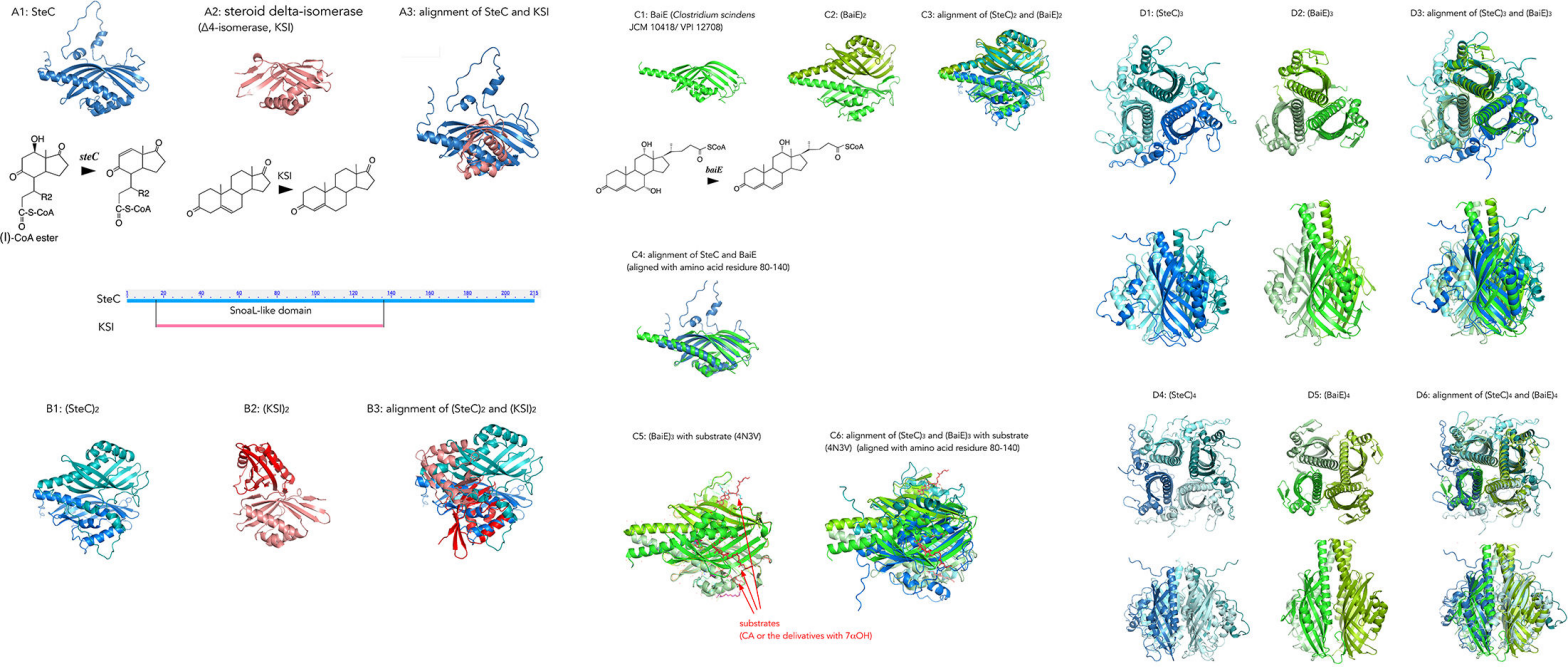
AlphaFold models of SteC (**A1**), ketosteroid ∆4-isomerase (KSI) (**A2**), alignment of SteC and KSI (RMSD = 1.27 Å over 39 cα atoms) (**A3**), dimeric structures (SteC)_2_ (**B1**) and (KSI)_2_ (**B2**), alignment of (SteC)_2_ and (KSI)_2_ (**B3**), BaiE, bile acid 7α-dehydratase of *Clostridium scindens* JCM 10418/ VPI 12708 (**C1**) (PDB: 4N3V) (54), (BaiE)_2_ (**C2**), alignment of (SteC)_2_ and (BaiE)_2_ (RMSD = 5.66 Å over 96 Cα atoms) (**C3**), alignment of amino acid residues 80–140 of (SteC)_2_ and (BaiE)_2_ (RMSD = 2.67 Å over 32 Cα atoms) (**C4**), (BaiE)_3_ with substrates (PDB: 4N3V) (**C5**), alignment of (SteC)_3_ and (BaiE)_3_ (PDB: 4N3V) (**C6**), trimeric structures of (SteC)_3_ (**D1**), (BaiE)_3_ (**D2**), and alignment of (SteC)_3_ and (BaiE)_3_ from different angles (**D3**), tetrameric structures (SteC)_4_ (**D4**) and (BaiE)_4_ (**D5**), and alignment of (SteC)_4_ and (BaiE)_4_ (**D6**). expected position errors and an alignment of five top models are shown in Fig. S8A through F.

SteC, an NTF2-family protein, shares this classification only with ketosteroid Δ⁴-isomerase (KSI) among TA441 steroid-degrading enzymes. Monomer alignment revealed similarity limited to the β-sheet region (approximately residues 20–160 in SteC) (Fig. 8A1 through A3; RMSD = 1.265 Å over 39 Cα atoms). Bacterial NTF2-family proteins fall into two major groups: catalytically active dimers, such as KSI, and catalytically inactive monomers (53). Because KSI forms a dimer and AlphaFold strongly predicted dimerization for SteC (Fig. S8A1 and A3, B1 and B3), we compared their dimer models; however, their overall architectures were distinct (Fig. 8B1 through B3; RMSD = 1.167 Å over 38 Cα atoms). No other TA441 steroid-degrading enzyme exhibited similarity to SteC. A broader search identified bile acid 7α-dehydratase BaiE from *Clostridium scindens* JCM 10418/VPI 12708 (formerly *Eubacterium* sp. VPI 12708) (54) as structurally similar (Fig. 8C1 and C2; Fig. S8A2 and B2). However, superposition of (SteC)₂ and (BaiE)₂ yielded an RMSD of 5.687 Å over 197 Cα atoms (Fig. 8C3). ClustalW alignment showed higher sequence homology within residues ~80–140 (Fig. S8C). Alignment of the monomers on residues 80–140 from both proteins gave an RMSD of 2.673 Å over 32 Cα atoms (Fig. 8C4).

Members of *Clostridium* are known to induce intestinal regulatory T cells and play major roles in mucosal immunity (55–57). BaiE is essential for conversion of primary to secondary bile acids (58), the latter being associated with increased colorectal cancer risk (59). Although both SteC and BaiE are NTF2-family proteins containing SnoaL-like domain functioning as dehydratases on hydroxyl groups of steroid rings—SteC on the C-ring and BaiE on the B-ring—they share only 28% amino acid identity (60–62). Crystal analysis showed that BaiE forms a trimer (54), and a trimeric crystal structure in complex with substrate is available (PDB: 4N3V) (54) (Fig. 8C5). Comparison of trimeric models of SteC and BaiE suggested that the β-sheet region (~residues 80–140) may constitute the substrate-binding site in SteC (Fig. 8C6). Superimposition of AlphaFold-predicted trimer models of (SteC)₃ and (BaiE)₃ showed notable structural similarity, while that of (KSI)₃ suggested that KSI works as a dimer (Fig. 8D1 through D3; Expected position errors and alignments of top models are shown in Fig. S8D, F1 and F2).

AlphaFold modeling indicated SteC and BaiE also form stable tetramer structure and they exhibited almost the same predicted position errors in dimeric, trimeric, and tetrameric models (Fig. 8D4 through D6; Expected position errors and alignments of top models are shown in Fig. S8E, F3 and F4). Superimposition of corresponding oligomeric models yielded similar RMSD values: monomer (5.662 Å over 96 Cα atoms), dimer (5.687 Å over 197 Cα atoms), trimer (5.699 Å over 300 Cα atoms), and tetramer (5.979 Å over 406 Cα atoms). The reason for this behavior remains unclear; however, such structural flexibility may enable stable interactions with diverse steroid substrates.

## DISCUSSION

In this study, we identified the dehydrase responsible for converting the 7α-hydroxyl group of **III**-CoA ester to **IV**-CoA ester and the 9α-hydrogenase responsible for converting 9,17-DOHNA-CoA ester to **III**-CoA ester, which had been predicted but not previously identified, and we designated these enzymes ScdH and ScdB, respectively. As a result, all reactions occurring at the beginning of B-, C-, and D-ring degradation—specifically, those before the initiation of β-oxidation of the opened B-ring—were fully elucidated, and the degradation of the sterane structure up to D-ring cleavage, including the removal of hydroxyl groups at positions 3, 7, and 12, has now been completely clarified.

Although *scdH* and *scdB* are essential genes for B-, C-, and D-ring degradation, they are not located within the BCD-ring degradation gene cluster *tesB* through *tesR*; instead, they are encoded adjacent to *chsH2E1H1ltp2chsE2*, the gene set responsible for degradation of the isopropyl residue at C17 in C17-side chain degradation of cholic acid (ORF38 and 39 in Fig. 1). The localization of *scdB* and *scdH* near genes involved in C17 side-chain degradation may reflect a requirement for coordinated expression of downstream enzymes with side-chain–degrading enzymes, enabling efficient and uninterrupted progression of bile acid degradation.

On the opposite side of *chsH2E1H1ltp2chsE2*, 5.7 kb upstream, genes encoding 3α-dehydrogenase and KSI are located, and approximately 38 kb farther upstream lie *steABCD*, which are required for removal of the 12α-hydroxyl group, along with the BCD-ring degradation gene cluster *tesB* through *tesR* (Fig. 1). To date, the major aerobic steroid degradation pathway appears to be largely conserved among bacteria, including *M. tuberculosis* (29). Thus, elucidating steroid degradation in *Comamonas,* an opportunistic bacterium ubiquitous in the environment, is expected to provide insight into diverse bacteria–host interactions mediated by steroids.

Using AlphaFold-based structural analysis, we found that many ketone/hydroxyl-converting hydrogenases/dehydrogenases involved in steroid degradation in TA441 are SDR-family oxidoreductases that adopt Rossmann-like α/β/α folds and function predominantly as dimers. In contrast, our analysis suggested that some enzymes may exist as distinct and stable three-dimensional conformers associated with the stereochemistry of their substrates. These structural variations may reflect evolutionary adaptations enabling single enzymes to accommodate a wider range of steroid substrates.

Furthermore, the dehydratase SteC, which removes the C12β-hydroxyl group from derivatives of 9,17-DOHNA, was found to have a three-dimensional structure strikingly similar to that of BaiE, a bile-acid 7α-dehydratase from obligate anaerobic Gram-positive bacteria *Clostridium scindens* JCM 10418/VPI 12708. The amino acid identity between SteC and BaiE is only ~28%, and conventional homology searches do not detect this similarity. BaiE plays a key role in the conversion of primary to secondary bile acids by gut bacteria. The finding that *C. testosteroni* possesses a structurally analogous enzyme capable of dehydrating hydroxyl groups on the steroid nucleus may provide new clues to understanding how bacteria have acquired the ability to degrade and modify diverse steroid compounds.

## MATERIALS AND METHODS

### Culture conditions

Mutant strains of *Comamonas testosteroni* TA441 were cultured at 30°C in a medium composed of equal volumes of Luria-Bertani (LB) medium and C medium, a mineral medium optimized for TA441 (9). This mixed medium was used because it allows mutants to accumulate intermediate compounds more efficiently than either C medium or LB medium alone (unpublished data). Lithocholic acid (LT) was added as a filter-sterilized solution in DMSO to a final concentration of 0.05% (w/v). LT—not lithocholate—was used because C medium serves as an effective buffer and maintains the pH around 7. Similarly, 3-(3-hydroxyphenyl)propionic acid (3HPP) was prepared in acetonitrile and added to the medium at a final concentration of 0.1% (w/v). Unless otherwise indicated, cultures were incubated for 7 days for LC/MS analysis. 3HPP was added 1 day after the start of incubation, as it inhibits the growth of TA441 when added at the beginning.

### Construction of deletion mutants, plasmids, and mutants for complementation experiments

To construct the ORF38-disrupted mutant (Table 1), an HpaI site was introduced into ORF38 on the pUC19-based plasmid pUC35-39 (63) (Table S1) using primers ORF38_Kmr and ORF38_KmrRC (Table S2). The kanamycin resistance gene (Kmr) was then inserted into the HpaI site, generating plasmid pUCORF38-Kmr (ORF38::Kmr). The plasmid was introduced into *C. testosteroni* TA441 via electroporation, and transformants were selected on LB plates containing kanamycin (400 µg/mL). Insertion of Kmr into ORF38 was confirmed by PCR using genomic DNA from the transformants. The ORF39-disrupted mutant was constructed similarly.

Plasmids for complementation experiments, such as pMFYMhpORF38 (Table S1), were constructed by amplifying DNA fragments containing the target ORF(s) and the Kmr gene using primers listed in Table S2. For example, primers MhpRPvuII_ORF38 and ORF38_KmrHR were used to amplify the ORF38 fragment, whereas primers ORF38_KmrH and Kmr-MFYPvuIIR were used to amplify the Kmr fragment. The amplified fragments were ligated with PvuII-digested pMFYMhpRA using the In-Fusion HD Cloning Kit.

### Ultra-high-performance liquid chromatography (UPLC)/MS

A 1 mL culture was extracted twice with an equal volume of ethyl acetate under acidic conditions (adjusted to pH 2 with HCl). The ethyl acetate layer was collected, evaporated to dryness, and dissolved in 1 mL methanol. A 5 µL aliquot was injected into a UPLC/MS system (Waters Acquity UPLC H-Class–QDa; Waters, Milford, MA, USA).

### Reverse-phase liquid chromatography with tandem mass spectrometry (LC/MS/MS)

Reverse-phase LC/MS/MS analyses (Fig. S2 and S3) were performed by injecting 2 µL of samples prepared as described above for HPLC/MS. An Agilent 1100 HPLC system (Agilent, CA, USA) coupled to a 4000 QTRAP MS/MS detector (AB Sciex, Framingham, MA, USA) was used in negative ion mode with an L-column2 ODS (1.5 × 150 mm) Type L2-C18, 5 µm, 12 nm (GL Science, Tokyo, Japan). Elution was performed with 90% solution A (H₂O:formic acid = 100:0.1) and 10% acetonitrile for 1 min, followed by a linear gradient to 20% solution A and 80% acetonitrile over 7 min, maintained for 2 min. The flow rate was 0.2 mL/min. MS/MS conditions were as follows: ion source temperature, 450°C; spray needle voltage, –4.5 kV; sheath gas pressures, 60 (gas 1) and 70 (gas 2); curtain gas, 15. Collision energy was 20 V, and ions were detected by the Q3 detector.

## Data Availability

The author affirms that materials and data reasonably requested by others will be made available from a publicly accessible collection or provided in a timely manner at reasonable cost and in limited quantities to members of the scientific community for noncommercial purposes.

## SUPPLEMENTAL MATERIAL

Supplemental material aem.02331-25-s0001.pdf Fig. S1 to S8; Tables S1 and S2.

ASM does not own the copyrights to Supplemental Material that may be linked to, or accessed through, an article. The authors have granted ASM a non-exclusive, world-wide license to publish the Supplemental Material files. Please contact the corresponding author directly for reuse.
