# Supplementary material for: Identification of the C9-hydrogenase for 9,17-dioxo-1,2,3,4,10,19-hexanorandrostan-5-oic acid (9,17-DOHNA) and the 7α-dehydratase essential for initiating β-oxidation of the B-, C-, and D-rings in steroid degradation by Comamonas testosteroni TA441
Source: Appl Environ Microbiol. 2026 Apr 3;92(4):e02331-25. doi: 10.1128/aem.02331-25 (PMC13101464; doi:10.1128/aem.02331-25)
Supplement: Supplemental material — Fig. S1 to S8; Tables S1 and S2. [file aem.02331-25-s0001.pdf]

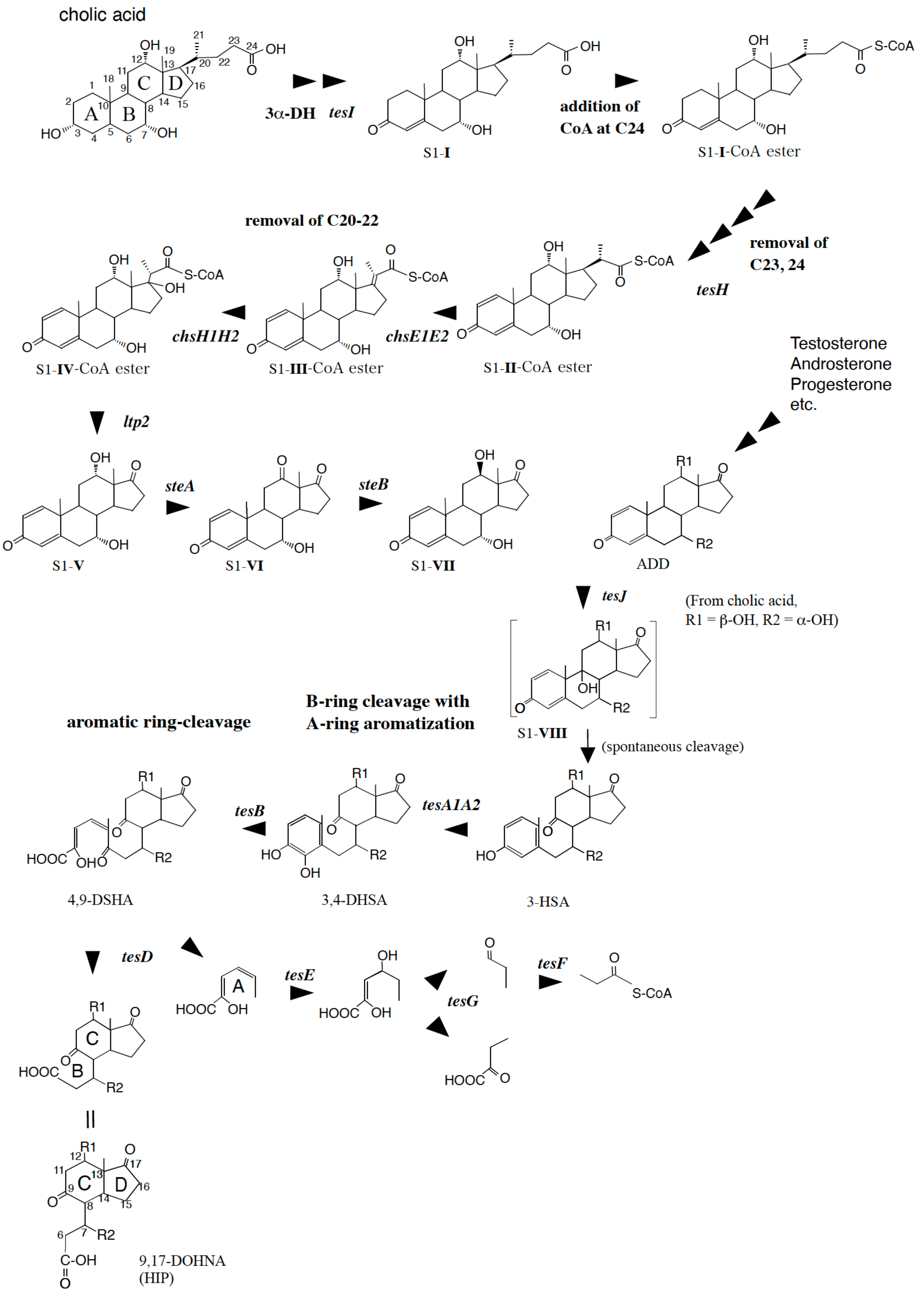
cholic acid
3α-DH tesI
S1-I
addition of
CoA at C24
S1-I-CoA ester
removal of
C23, 24
tesH
S1-II-CoA ester
chsE1E2
S1-III-CoA ester
chsH1H2
S1-IV-CoA ester
removal of C20-22
ltp2
S1-V
steA
S1-VI
steB
S1-VII
Testosterone
Androsterone
Progesterone
etc.
ADD
tesJ
(From cholic acid,
R1 = β-OH, R2 = α-OH)
S1-VIII
(spontaneous cleavage)
3-HSA
tesA1A2
3,4-DHSA
B-ring cleavage with
A-ring aromatization
tesB
4,9-DSHA
aromatic ring-cleavage
tesD
tesE
tesG
tesF
S-CoA
9,17-DOHNA
(HIP)

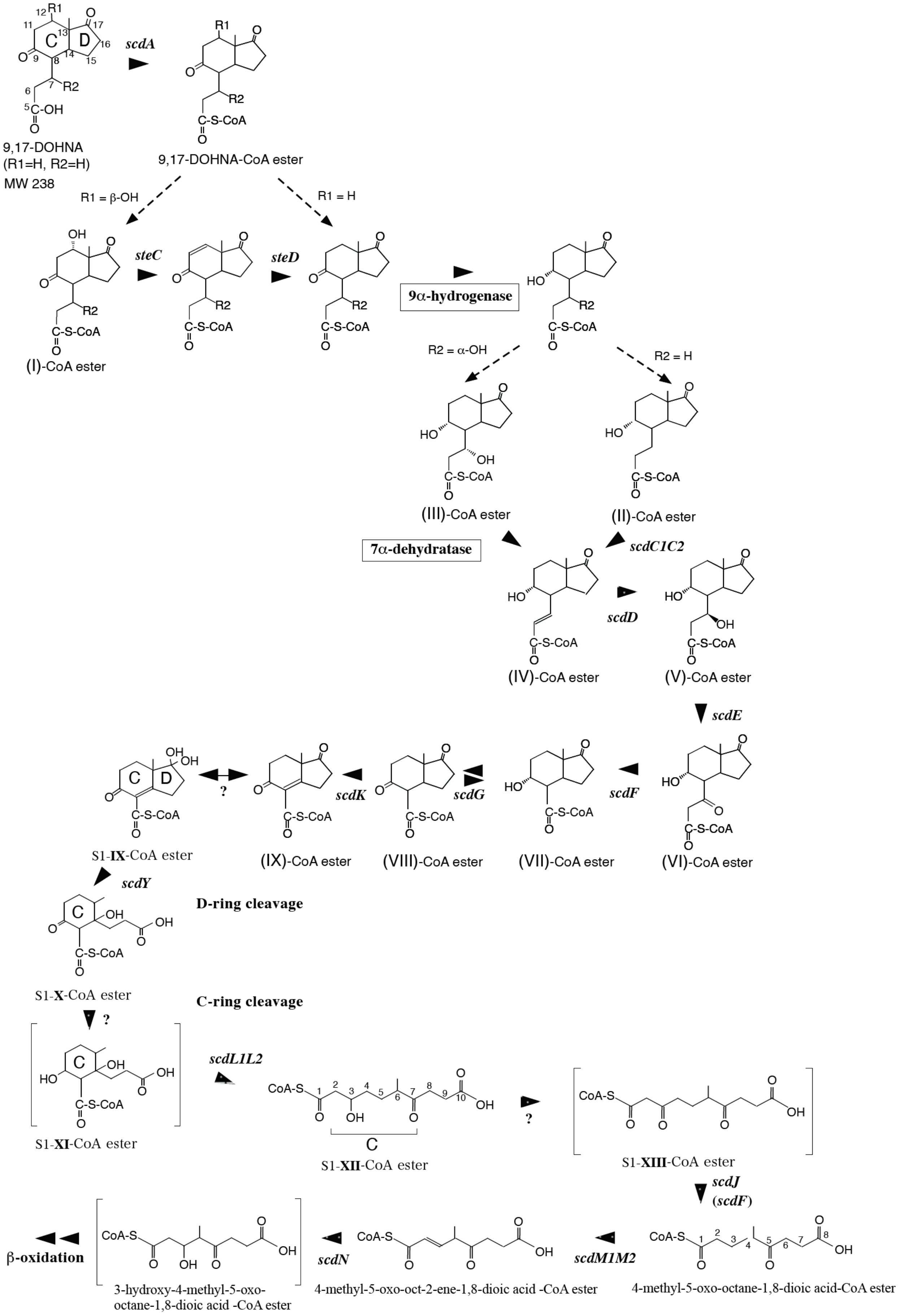
9,17-DOHNA
(R1=H, R2=H)
MW 238
scdA
9,17-DOHNA-CoA ester
R1 = β-OH
R1 = H
steC
steD
9α-hydrogenase
(I)-CoA ester
R2 = α-OH
R2 = H
(III)-CoA ester
(II)-CoA ester
7α-dehydratase
scdC1C2
scdD
(IV)-CoA ester
(V)-CoA ester
scdE
scdK
scdG
scdF
S1-IX-CoA ester
(IX)-CoA ester
(VIII)-CoA ester
(VII)-CoA ester
(VI)-CoA ester
scdY
D-ring cleavage
S1-X-CoA ester
C-ring cleavage
scdL1L2
S1-XI-CoA ester
S1-XII-CoA ester
S1-XIII-CoA ester
scdJ
(scdF)
β-oxidation
scdN
scdM1M2
3-hydroxy-4-methyl-5-oxo-octane-1,8-dioic acid -CoA ester
4-methyl-5-oxo-oct-2-ene-1,8-dioic acid -CoA ester
4-methyl-5-oxo-octane-1,8-dioic acid-CoA ester

Fig. S1 Overview of the steroid degradation pathway in *C. testosteroni* TA441. Most compounds were isolated and identified via NMR and mass spectrometry. Compounds with * were confirmed by mass spectrum and conversion experiments, while those in brackets are hypothetical.

Compounds are; (S1-**I**); 7α,12α-dihydroxy-3-oxo-5β-4-cholen-24-oic acid, (S1-**II**); 7α,12α-dihydroxy-3-oxo-1,4-pregnadine-20-carboxylic acid, (S1-**III**); 7α,12α-dihydroxy-3-oxo-1,4,17-pregnatrine-20-carboxylic acid, (S1-**IV**); 7α,12α,17-trihydroxy-3-oxo-1,4-pregnatriene-20-carboxylic acid, (S1-**V**); 7α,12α-dihydroxy-3,17-dioxo-1,4-androstadien, (S1-**VI**); 7α-hydroxy-3,12,17-trioxo-1,4-androstadien, (S1-**VII**); 7α,12β-dihydroxy-3,17-dioxo-1,4-androstadien, ADD; 3,17-dioxo-1,4-androstadien, (S1-**VIII**)(R1, R2=H); 9-hydroxy-3,17-dioxo-1,4-androstadien, 3-HSA (R1,R2 = H); 3-hydroxy-9,10-secoandrosta-1,3,5(10)-triene-9,17-dione, 3,4-DHSA (R1,R2 = H); 3,4-dihydroxy-9,10-secoandrosta-1,3,5(10)-triene-9,17-dione, 4,9-DSHA (R1, R2=H): 4,5-9,10-diseco-3-hydroxy-5,9,17-trioxoandrosta-1(10),2-dien-4-oic acid, 9,17-DOHNA; 9,17-dioxo-1,2,3,4,10,19-hexanorandrostan-5-oic acid (3aα-*H*-4α{3'-propionic acid}-7aβ-methylhexahydro-1,5-indanedione, HIP), (**I**); 12β-hydroxy-9,17-dioxo-1,2,3,4,10,19-hexanorandrostan-5-oic acid (R2=H) , (**II**); 9α-hydroxy-17-oxo-1,2,3,4,10,19-hexanorandrostan-5-oic acid, (**III**); 7α,9α-dihydroxy-17-oxo-1,2,3,4,10,19-hexanorandrostan-5-oic acid, (**IV**); 9α-hydroxy-17-oxo-1,2,3,4,10,19-hexanorandrost-6-en-5-oic acid, (**V**); 7β,9α-dihydroxy-17-oxo-1,2,3,4,10,19-hexanorandrostan-5-oic acid, (**VI**); 9α-hydroxy-7,17-dioxo-1,2,3,4,10,19- hexanorandrostan-5-oic acid, (**VII**); 9α-hydroxy-17-oxo-1,2,3,4,5,6,10,19-octanorandrostan-7-oic acid, (**VIII**); 9,17-dioxo-1,2,3,4,5,6,10,19-octanorandrostan-7-oic acid, (**IX**); 9,17-dioxo-1,2,3,4,5,6,10,19-octanorandrost-8(14)-en-7-oic acid, (S1-**IX**); 17-dihydroxy-9-oxo-1,2,3,4,5,6,10,19-octanorandrost-8(14)-en-7-oic acid, (S1-**X**); 14-hydroxy-9-oxo-1,2,3,4,5,6,10,19-octanor-13,17-secoandrostane-7,17-dioic acid, (S1-**XI**); 9,14-dihydroxy-1,2,3,4,5,6,10,19-octanor-13,17-secoandrostane-7,17-dioic acid, (S1-**XII**); 3β-hydroxy-6-methyl-7-oxo-decane-1,10-dioic acid, and (S1-**XIII**); 6-methyl-3,7-dioxo-decane-1,10-dioic acid.

Enzymes are; **3α-DH** (3α-dehydrogenase for compounds with sterane structure); **ChsE1E2** (Δ17(20) dehydrogenase for 3-oxo-4,17-pregnadiene-20-carboxyl-CoA), **ChsH1H2** (C17 hydratase for 3-oxo-4-pregnene-20-carboxyl-CoA), **Ltp2** (aldolase for 17-hydroxy-3-oxo-4-pregnene-20-carboxyl-CoA), **SteA** (dehydrogenase for 12α-OH to 12-ketone), **SteB** (hydrogenase for 12-ketone to 12β-OH), **TesH** (Δ1-dehydrogenase), **TesI** (Δ4-dehydrogenase), **TesJ** (ADD-hydroxylase at C9), **TesA1A2** (3-HSA-hydroxylase at C4), **TesB** (*meta*-cleavage enzyme for 3.4-DHSA), **TesD** (4.9-DSHA -hydrolase), **TesE** ((2*Z*,4*Z*)-2-hydroxyhexa-2,4-dienoic acid-hydratase), **TesF** (aldolase), **TesG** (acetoaldehyde dehydrogenase), **SteC** (dehydradase for 12β-OH to produce a double at C10(12)), **SteD** (reductase for a double at C10(12) to a single bond), **ScdA** (CoA-transferase for 9,17-dioxo-1,2,3,4,10,19-hexanorandrostan-5-oic acid), **ScdG** (hydrogenase for 9-OH of 9α-hydroxy-17-oxo-1,2,3,4,5,6,10,19-octanorandrostan-7-oic acid-CoA ester), **ScdC1C2** (Δ6-dehydrogenase for 9α-hydroxy-17-oxo-1,2,3,4,10,19-hexanorandrostan-5-oic acid-CoA ester), **ScdD** (9α-hydroxy-17-oxo-1,2,3,4,10,19-hexanorandrost-6-en-5-oic acid -CoA ester Δ6-hydratase), **ScdE** (7β,9α-dihydroxy-17-oxo-1,2,3,4,10,19-hexanorandrostan-5-oic acid-CoA ester dehydrogenase at C7), **ScdF** (9α-hydroxy-7,17-dioxo-1,2,3,4,10,19-hexanorandrostan-5-oic acid-CoA ester thiolase/CoA-transferase), **ScdK** (Δ8(14)-dehydrogenase for 9,17-dioxo-1,2,3,4,5,6,10,19-octanorandrostan-7-oic acid-CoA ester), **ScdY** (17-dihydroxy-9-oxo-1,2,3,4,5,6,10,19-octanorandrost-8(14)-en-7-oic acid-CoA ester hydratase), **ScdL1L2** (putative CoA-transferase/isomerase necessary for C-ring cleavage of 9,14-dihydroxy-1,2,3,4,5,6,10,19-octanor-13,17-secoandrostane-7,17-dioic acid-CoA ester or maybe C-ring cleavage of 14-hydroxy-9-oxo-1,2,3,4,5,6,10,19-octanor-13,17-secoandrostane-7,17-dioic acid-CoA ester), **ScdJ** (6-methyl-3,7-dioxo-decane-1,10-dioic acid-CoA ester thiolase/CoA-transferase), **ScdM1M2** (4-methyl-5-oxo-octane-1,8-dioic acid-CoA ester dehydrogenase), and **ScdN** (4-methyl-5-oxo-oct-2-ene-1,8-dioic acid -CoA ester hydratase). Genes for the C-, D-, and cleaved B-ring degradation are induced by positive regulator **TesR** with compounds having sterane structure.

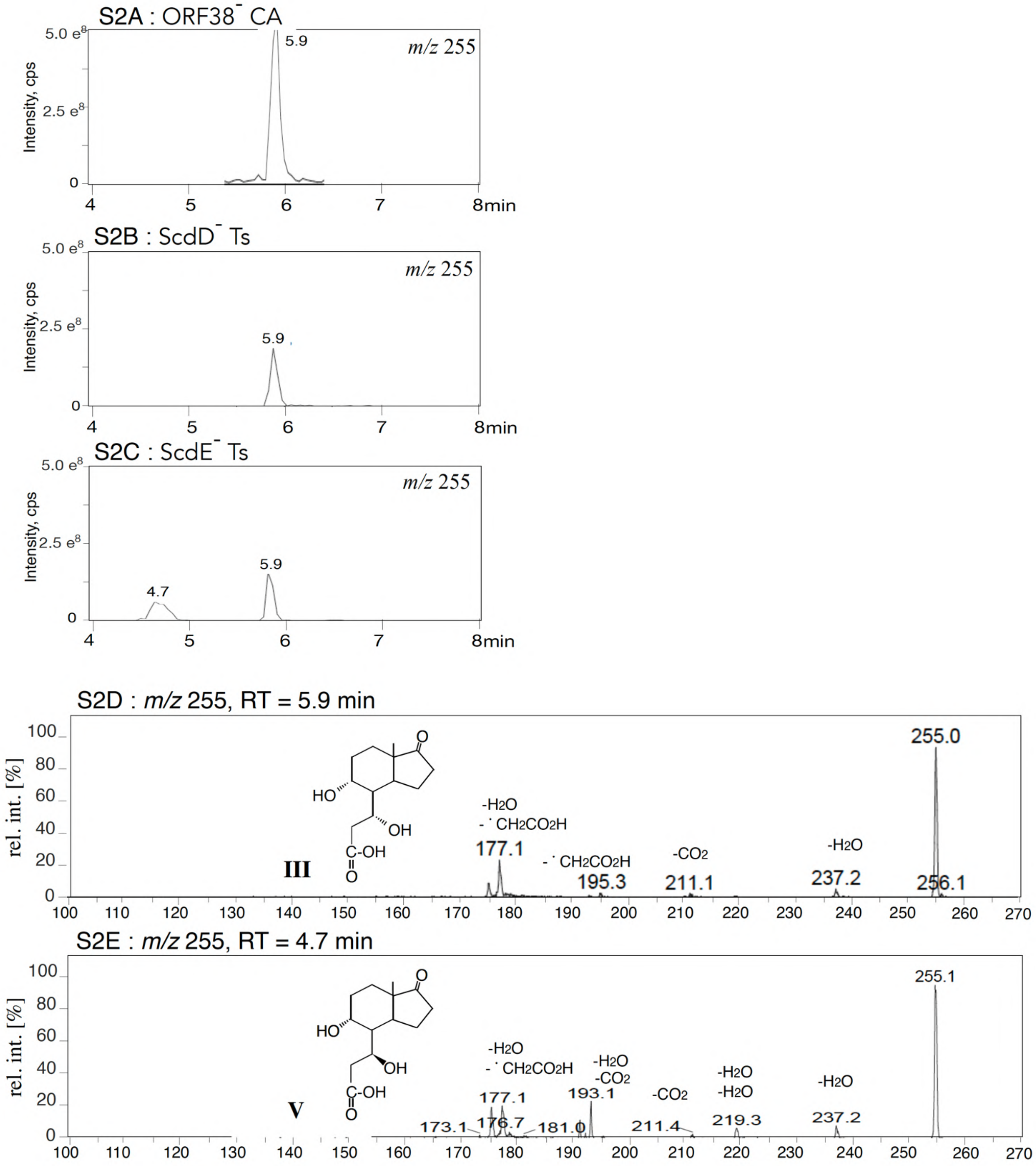

Fig. S2 Chromatograms of *m/z* 255 of LC/MS/MS analysis of the culture of the ORF38⁻ incubated with cholic acid (CA) (S2A), ScdD⁻ with testosterone (Ts) (S2B), ScdE⁻ with Ts (S2C), the Mass spectrums of the peak with *m/z* of 255 at around RT = 5.9 min (**III**) (S2D), and of the peak with *m/z* of 255 at around RT = 4.7 min (**V**) (S2E). Agilent 1100 HPLC (Agilent, CA) with a mass spectrometer, 4000 QTRAP MS/MS system (AB SCIEX, Framingham, MA, USA) in negative ion mode, was used with L-column2 ODS (1.5 × 150 mm) Type L2-C 18.5μm, 12mm (GL Science, Tokyo, Japan) and elution was carried out using 90% solution A (H_2O:HCOOH = 100:0.1) and 10% acetonitrile for 1min, followed by a linear gradient from 90% solution A and 10% acetonitrile to 20% solution A and 80% acetonitrile over 7 min, which was maintained for 2 min. The flow rate was 0.2 ml/min. The vertical axis indicates intensity (count/sec) and the horizontal axis indicates RT (min) in Mass chromatograms, and the vertical axis indicates relative intensity (%) and the horizontal axis indicates molecular weight in Mass spectrums.

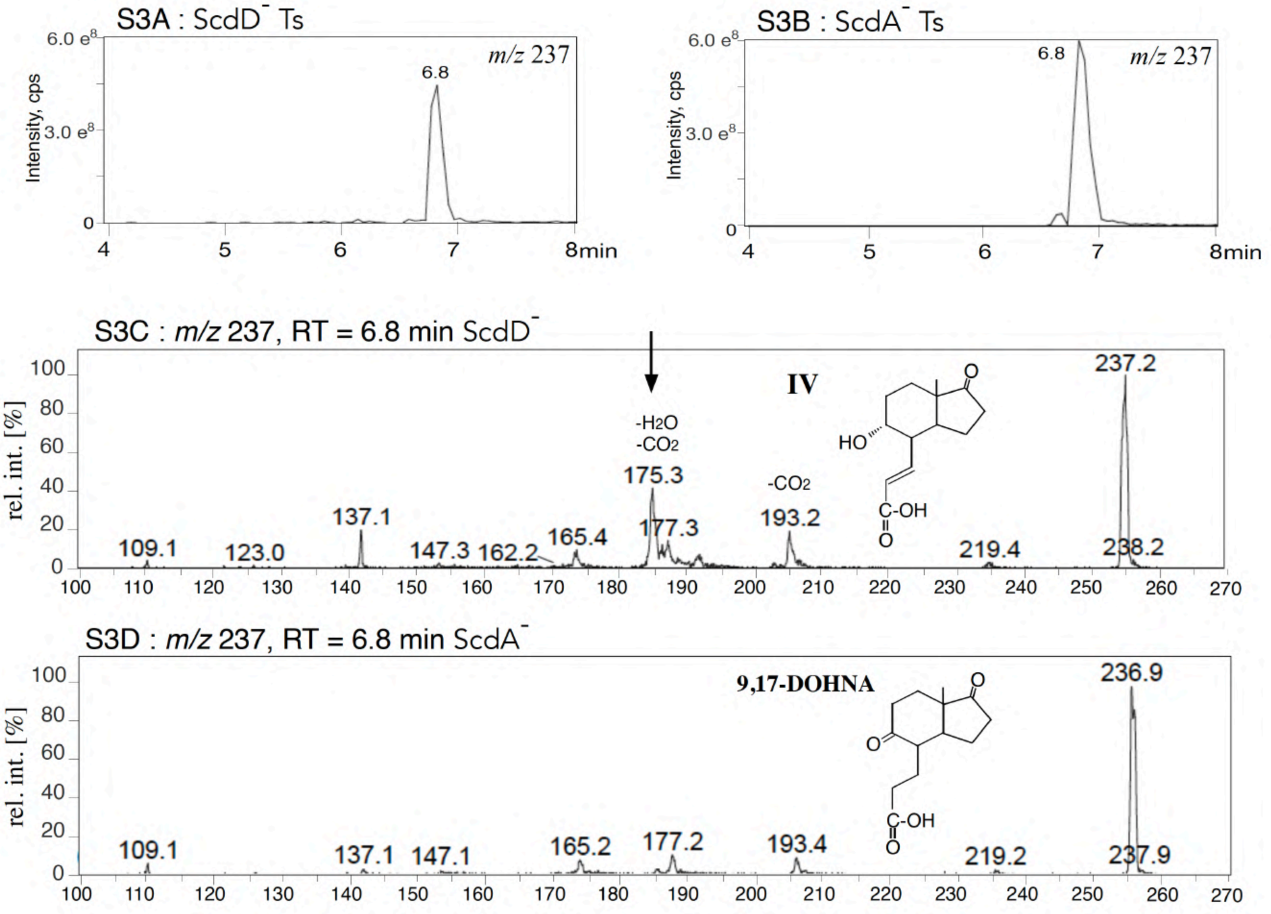

Fig. S3 Chromatograms of *m/z* 237 of LC/MS/MS analysis of the culture of the ScdD$^-$ (S3A) and ScdA$^-$ (S3B) incubated with Ts, and the Mass spectrum of the peak with *m/z* of 237 at around RT = 6.8 min in ScdD$^-$ culture (**IV**) (S3C), and in ScdA$^-$ culture (9,17-DOHNA) (S3D). Agilent 1100 HPLC (Agilent, CA) with a mass spectrometer, 4000 QTRAP MS/MS system (AB SCIEX, Framingham, MA, USA) in negative ion mode, was used with L-column2 ODS (1.5 × 150 mm) Type L2-C 18.5μm, 12mm (GL Science, Tokyo, Japan) and elution was carried out using 90% solution A (H_2O:HCOOH = 100:0.1) and 10% acetonitrile for 1 min, followed by a linear gradient from 90% solution A and 10% acetonitrile to 20% solution A and 80% acetonitrile over 7 min, which was maintained for 2 min. The flow rate was 0.2 ml/min. The vertical axis indicates intensity (count/sec) and the horizontal axis indicates RT (min) in Mass chromatograms, and the vertical axis indicates relative intensity (%) and the horizontal axis indicates molecular weight in Mass spectrums.

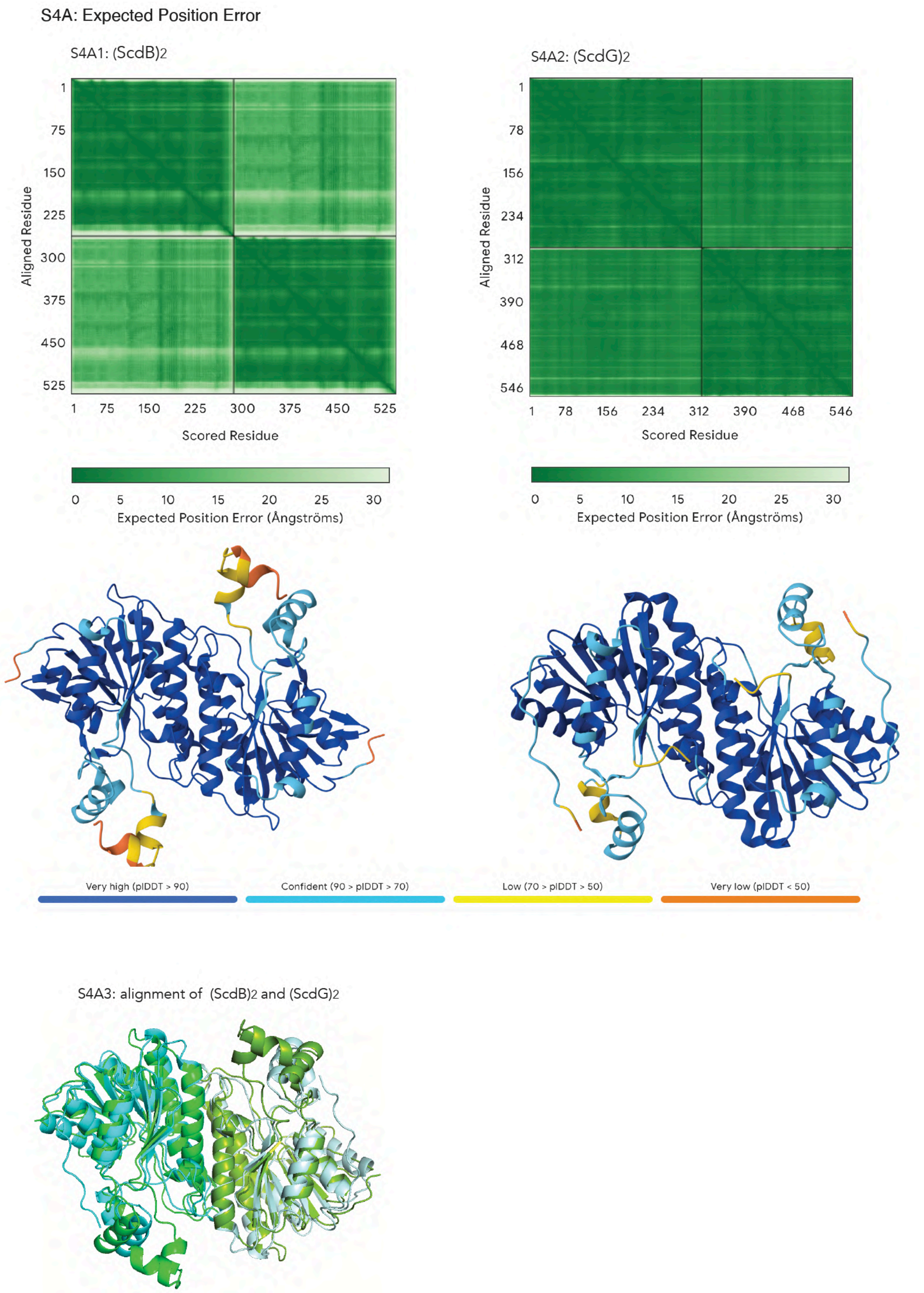

Fig. S4 **S4A:** Expected position errors of $(ScdB)_2$ (S4A1) and $(ScdG)_2$ (S4A2), and alignment of AlphaFold models of $(ScdB)_2$ and $(ScdG)_2$ (RMSD = 1.06 Å over 210 Cα atoms) (S4A3). In the expected position error plots, darker green indicates stronger predicted interactions between amino acid residues on the X- and Y-axes.

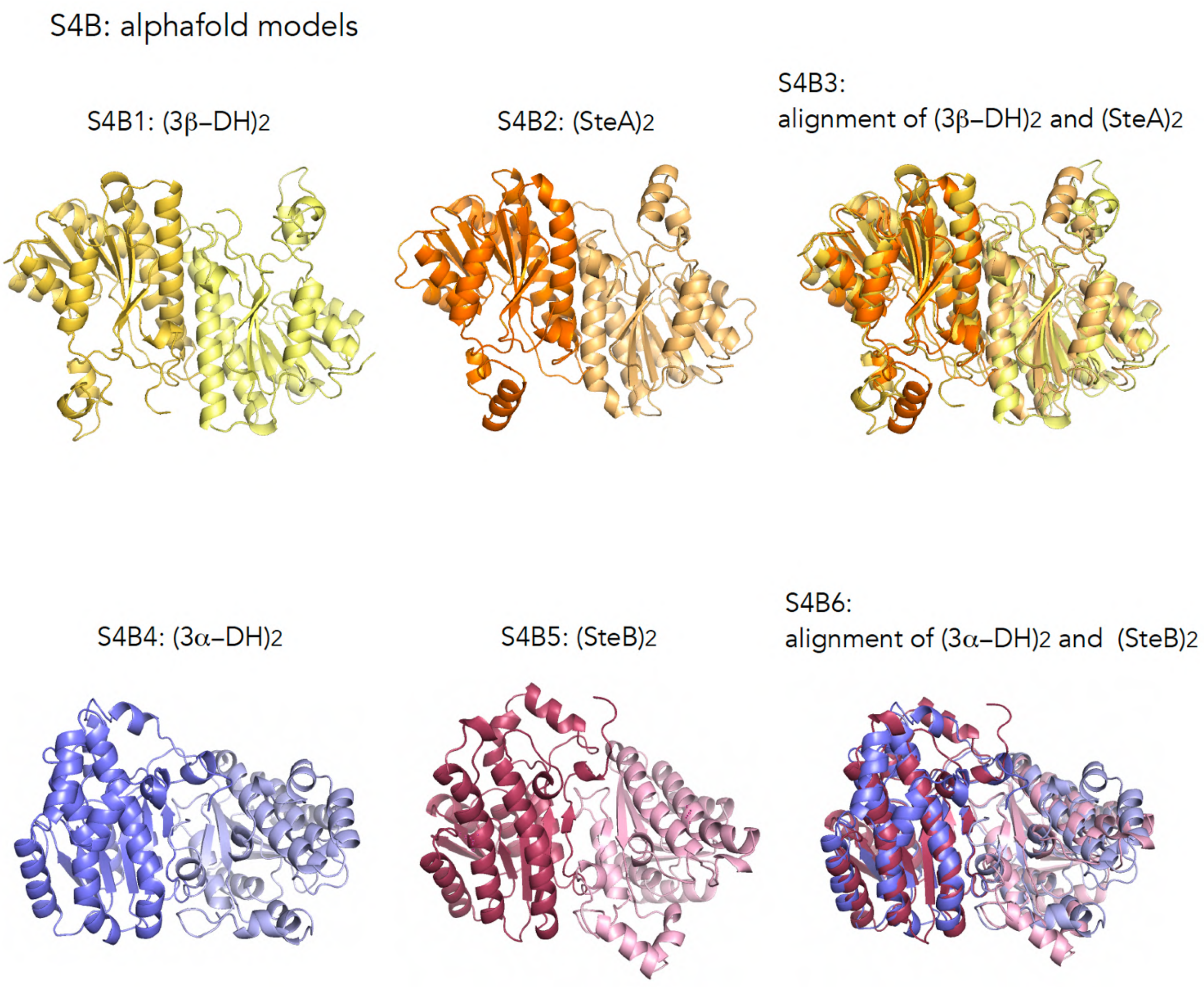

S4B: AlphaFold models of $(3\beta\text{-DH})_2$ (S4B1) and $(\text{SteA})_2$ (S4B2) with their alignment (RMSD = 2.03 Å over 330 Cα atoms) (S4B3), and AlphaFold models of $(3\alpha\text{-DH})_2$ (S4B4) and $(\text{SteB})_2$ (S4B5) with their alignment (RMSD = 1.83 Å over 260 Cα atoms) (S4B6).

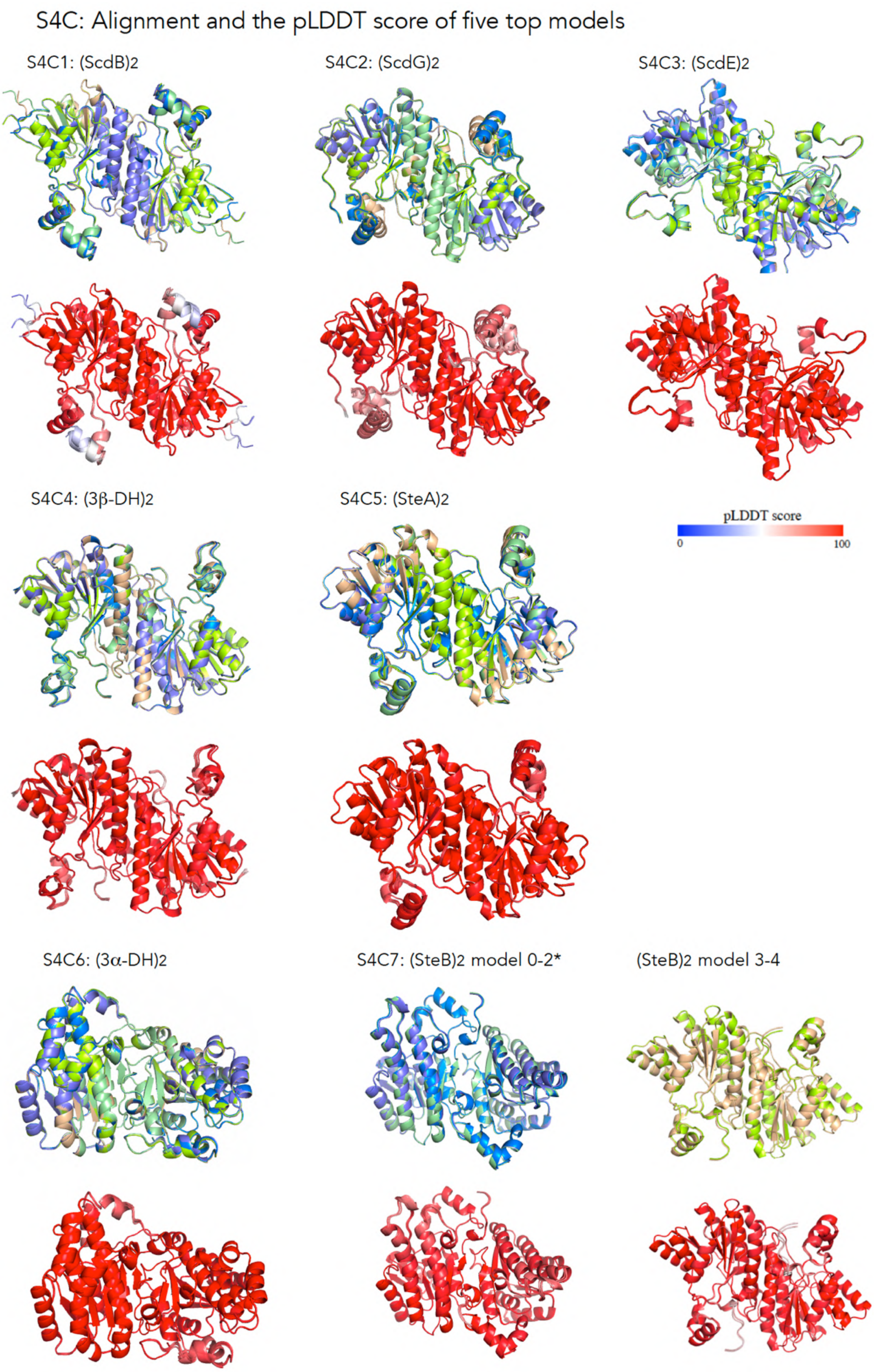

S4C: Alignment of the five top-ranked models and pLDDT scores of $(ScdB)_2$ (S4C1), $(ScdG)_2$ (S4C2), $(ScdE)_2$ (S4C3), $(3\beta\text{-DH})_2$ (S4C4), $(SteA)_2$ (S4C5), $(3\alpha\text{-DH})_2$ (S4C6), and $(SteB)_2$ (S4C7). Models 0–2 of $(SteB)_2$ were similar to the $(3\alpha\text{-DH})_2$ model, whereas Models 3 and 4 were similar to the $(ScdB)_2$ model. Color scheme: marine, model_0; slate, model_1; palegreen, model_2; limon, model_3; wheat, model_4.

S4D: Alignment of amino acid sequences of the hydrogenase/dehydrogenases

```
3alphaDH        ---------------------------MSIIVMSGCATGIGAATRKVLEAAGHQIV-GID   32
SteB            ---------------------MSKRLEGKIAFVTGGGSGIGAATAERFAQEGATVV-ICG   38
ScdE            VISRAQTGCGPALHSKQKGKEMG-ICNQRTVVITGAGGGLGRAYALAFAQEGANVV-IND   58
ScdB            -------------------MDTNKSFQDKVVLVTGGTKGIGKGIAQAFLAAGATVV-VCG   40
ScdG            ------MTSAPS-----YVAPHGLLKDKTVLVTAAAGAGIGFSAAKRAAEEGCRALFISD   49
SteA            -----------------------MRLAGKVAVVTGAGQGMGRAIAQRFADEGATVV-AVD   36
3betaDH         ---------------------MTNRLQGKVALVTGGASGVGLEVVKLLLGEGAKVA-FSD   38
                                               . :.   *:*          *       .

3alphaDH        VRDAEV---IADLSTAEGRK---------------QAIADVLAKCSKGMDGLVLCAGLGP   74
SteB            RRKQPLDEVVARIKAAGGSA--EAIVADVGNEAQFVGALEQTAKKHGSLDILVNNAMAYT   96
ScdE            IRAEAAQAVAEEVKAAGGKA--LANTGDITTVAGAQSILDAALAAFGDVQVIVNNAGVLR   116
ScdB            RQQPE---ALPSINGSSA----DFVACDVREAQAVKDMVDEVQRRHGRLDVLVNNAGGAP   93
ScdG            VHERRLAEAVEQIKKDTGLSQVFGKLCDVSKEEQVQALISEADSAMGGIDVLINNAGLGT   109
SteA            LNEEAARQTLE---GKSGKH--LARSSNVADSAAVDALFVEIREKLGTVDVLVNNAGVGS   91
3betaDH         INAAGQQLAAE-----LGE-RSMFVRHDVSSEADWTLVMAAVQRRLGTLNVLVNNAGILL   92
                 .               .                              :: ::  *

3alphaDH        QTRV-------LGNVVSVNYFGATELMDAFLPLLKQGR-----Q-PAAVVISSVASAHLA   121
SteB            WGGIDAMTTADWHANFSTSVDGTFWGTRTALKLMG--A-----KGGSIVNISSIC-----   144
ScdE            DRMFLSLSEEDWDMVMRVHLRGHFCMAKVFGGYWRDQKKAGKDVDARIINTSSGA-----   171
ScdB            HVDAATVSPRFHESVLRLNLFSTLHASQAANAVMQQQD-----GGGVIVCIGSIS-----   143
ScdG            SCKVVDMGDDEWSKVIDITLTGTFRMTRAALKLMQ-PR-----GKGVIVNNASVL-----   158
SteA            VDQFADIPDATWERVIGVNLNGAFYCARAAVKQMQ-EG-----KGGAIVNISSTS-----   140
3betaDH         PGDMETGRLEDFSRLLKINTESVFIGCQQGIAAMK--E-----TGGSIINMASVS-----   140
                               .     .                          :  .*

3alphaDH        FDKNPLAPALEAGEEAKARAIVEQAGEQGGNLAYAGSKNALTVAVRKRTAAWGEAG---V   178
SteB            -----------------------GTLGTPFMSGYSAAKAAIINFSRAAAAEGAAAG---I   178
ScdE            -----------------------GLQGSIGQSNYAAAKAGIAGLTLVQAAELARYG---I   205
ScdB            -----------------------ALRPSPGTAAYGAAKAAVLSLVSSLAVEWA-PK---V   176
ScdG            -----------------------GWRAQTEQAHYAAAKAGVMALTRCSALEAAEFG---I   192
SteA            -----------------------AVSGD-GPAHYCASKAALMGLTRGMAKELASKK---I   173
3betaDH         -----------------------SWLPIEQYAGYSASKAAVSALTRAAALSCRKQGYAIR   177
                                                 * .:* .:       :

3alphaDH        RLNTIAPGATETPLLQAGLQDPRYGESI--AKFVPPMGRRAEPSEMASVIAFLMSPAASY   236
SteB            RVNVVIPAVVETPATAGMLADEASRKNT---EKLIPMGRVGQSNELANAVLFLASEEASY   235
ScdE            TANCLAPAART-SMTEGAMPDM-----V---KKPESGFDVWDPMNVASIVVWLGSTQSRH   256
ScdB            RVVAVSPGLVRTEQSHLHFGDEAGIASV---AGTIPAGRLAEPQDIANACLYVASENASY   233
ScdG            RINAVAPSIAIHAFLKKSASEELL-AKL---SEKEAFGRGAEPWEVANVMIFLASDYSSY   248
SteA            RVNTLVPGPTNTPMMQGIPQEWA--DAI---IAGVPMGRMAEPEDIAKVAVFLASDDSGF   228
3betaDH         RVNSIHPDGIYTPMMQASLPKGVSKEMVLHDPKLNRAGRAYMPERIAQLVLFLASDESSV   237
                    : *             .                        :*.   :: *  :

3alphaDH        VHGAQIVIDGGIDAVMRPTQF-----------------------------------   257
SteB            VTGAALPVDGGRSAVLVTAL------------------------------------   255
ScdE            VTGRCFEAKGGELSVADGWFTGKINDKQARWEPAELSGVVDQLIAEGKAPQKVYGT   312
ScdB            MSGTNLLLHGGGERPAFLSASNSESVQQKH--------------------------   263
ScdG            MTGEVVSVSSQRA-------------------------------------------   261
SteA            VTGQNVAVNGGSAFL-----------------------------------------   243
3betaDH         MSGSELHADNSILGMGL---------------------------------------   254
                : *  .   .
```

S4D: Clustal multiple amino acid sequence alignment of 3α-DH, SteB, ScdE, ScdB, ScdG, SteA, and 3β-DH. Amino acids highlighted in green boxes are conserved in all enzymes, whereas those in blue boxes are conserved in two or three enzymes containing 3α-DH and SteB. Symbols: “*”, identical; “:”, highly similar; “.”, similar.

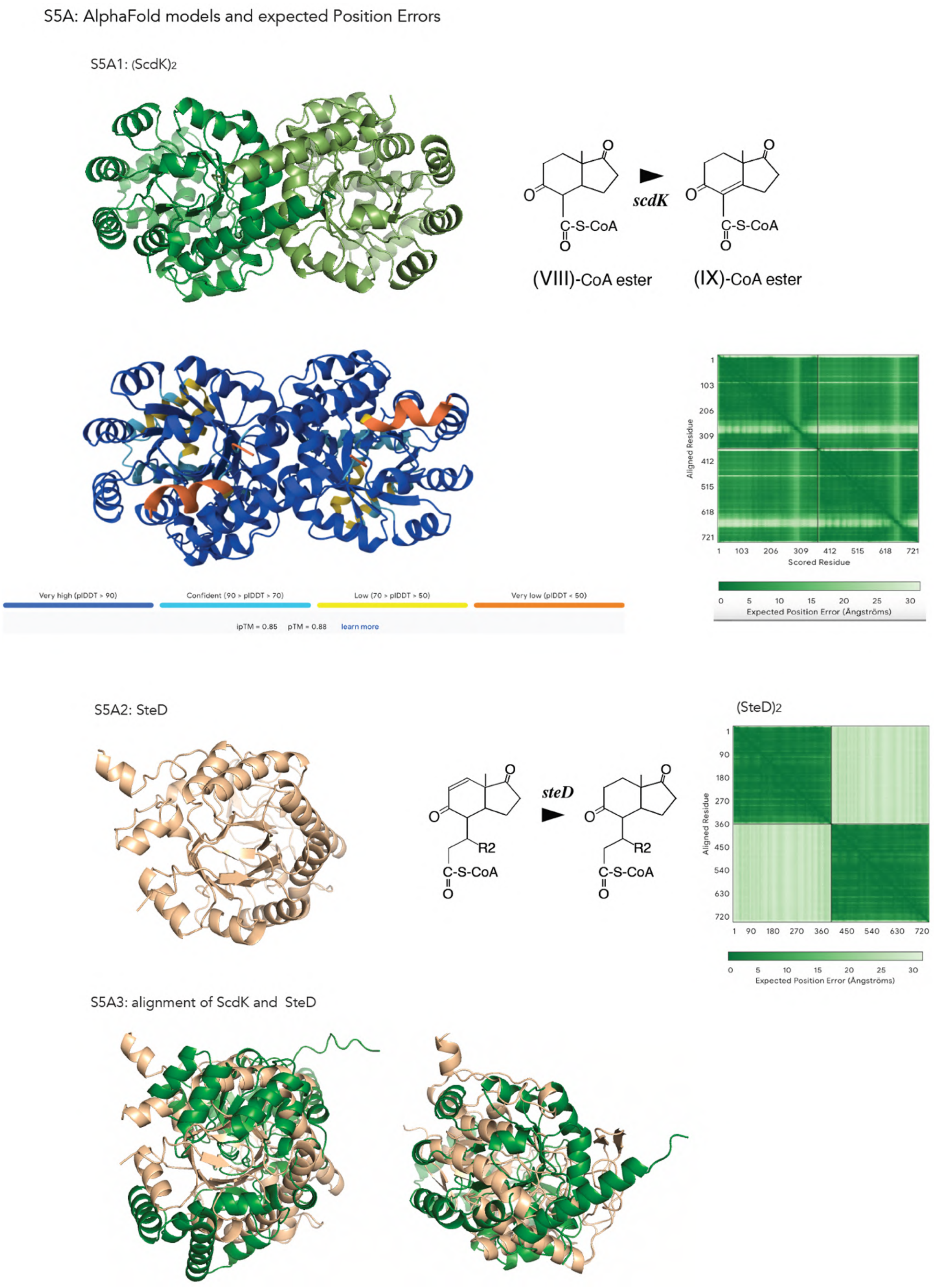

Fig. S5 **S5A:** AlphaFold models and expected position errors of (ScdK)₂, C8(14) dehydrogenase for the **VIII**-CoA ester (S5A1), and SteD, C10(12) hydrogenase for the 9,17-dioxo-1,2,3,4,5,6,10,19-hexanorandrost-10-ene-5-oic acid–CoA ester (S5A2), with alignment of ScdK and SteD (S5A3). In the expected position error plots, darker green indicates stronger predicted interactions between amino acid residues on the X- and Y-axes.

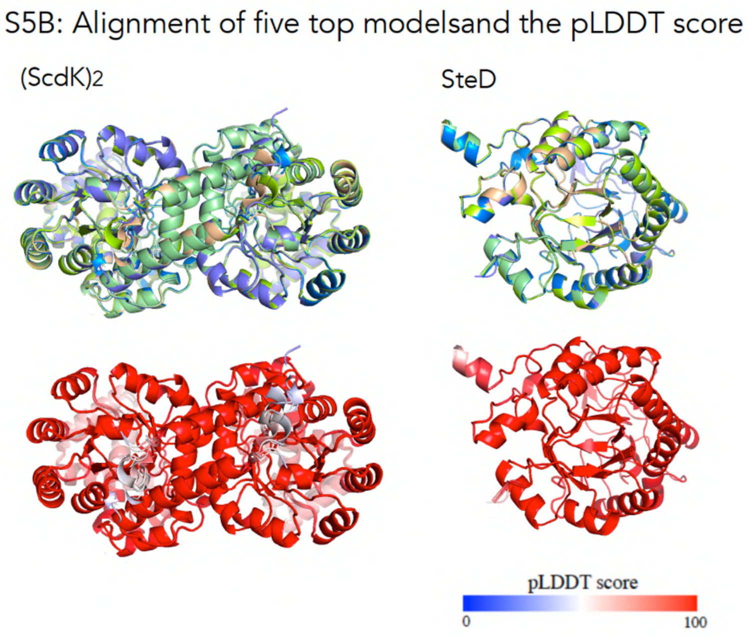

S5B: Alignment of the five top-ranked models and pLDDT scores of $(ScdK)_2$ and SteD. Color scheme: marine, model_0; slate, model_1; palegreen, model_2; limon, model_3; wheat, model_4.

S6A: Expected Position Error

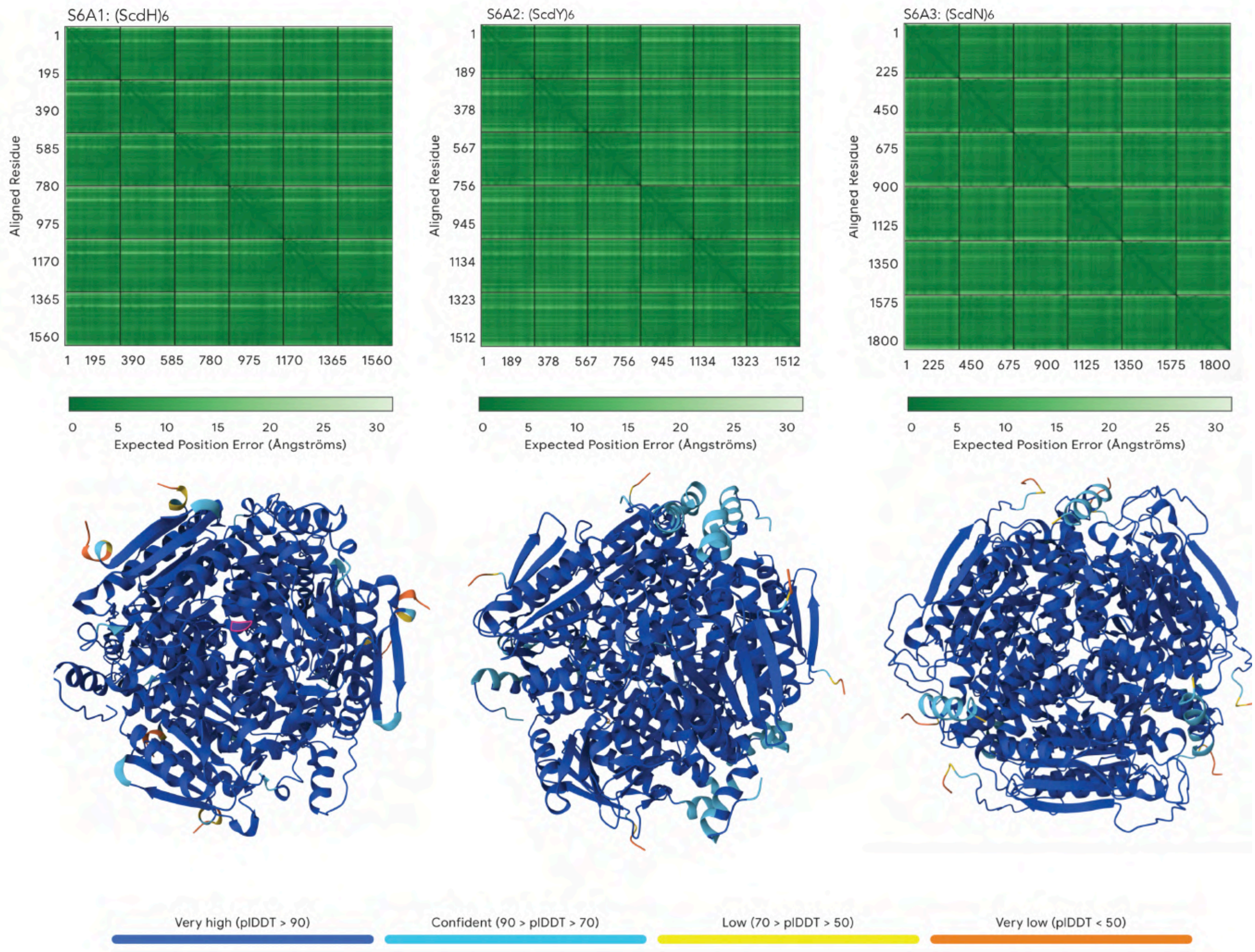

S6A4: Alignment of (ScdH)6 + (ScdY)6

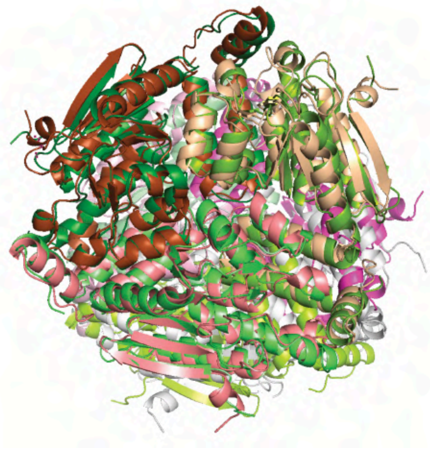

S6A5: Alignment of amino acid sequences of ScdH, enoyl-CoA hydratase from *Rattus norvegicus* (1DUB), ScdY, and ScdN

```
ScdH            ----MSASLSAIENMDTAQRELLVRHQDHGVAIVQLNRPEATNALSLSLQAALSRTFAEL   56
1DUB            -------------GANFQYIITEKKGKNSSVGLIQLNRPKALNALCNGLIEELNQALETF   48
ScdY            ----------------MSTQQFHSNIHDNGVAELVIDR-APVNALNAAGWNGLAREIQAL   44
ScdN            MGDASSEFVTREDKAVYETDEPVLYSVDNGIATVTMNRPTFNNVQNSQMTYALDAAFRKA   60
                                           : .:. : ::*    *.        *   :

ScdH            SADASVRCIVLTGGDKVFAAGGDIKSMDS---------------------CGPIEIMKRH   96
1DUB            EEDPAVGAIVLTGGEKAFAAGADIKEMQN---------------------RTFQDCYSGK   86
ScdY            GDRPEVRVIVIRAENRGFCAGVDIKELAED--------------------DKLILDVNAG   83
ScdN            TDDDSVKVIVLRGEGKHFSAGHDIGTPGRDINKSFDRAHLWWDHTNKPGGEQLFAREQEV  120
                     *  **: .  : *.** **                                 .

ScdH            TERVWAPIEKCPKPIIAAVCGYAFGGGAELAMHCDIIIAGQGASFAQPEIRIGIMPGIGG  156
1DUB            FLSHWDHITRIKKPVIAAVNGYALGGGCELAMMCDIIYAGEKAQFGQPEILLGTIPGAGG  146
ScdY            NYATFKAVHLNKVPVITAVHGFVLGGGIGICGASDIVIAAEDATFGLPEVDRGAMG---G  140
ScdN            YLGMCRRWREIPKPMIAMVQGACVAGGLMLAWVCDLIVASDDAFFQDPVVRMGIPG--VE  158
                             *:*: * *  ..**  :.  .*:: *.: * *  * :  *

ScdH            TQRLVRAVGKFQAMRILLTGKPVSADEAYAMGLVSLVCADDQVIPEALKMAKLIANMPPL  216
1DUB            TQRLTRAVGKSLAMEMVLTGDRISAQDAKQAGLVSKIFPVETLVEEAIQCAEKIANNSKI  206
ScdY            AAHLQRMFGVQKTRYLFFTGEMIGAAEALRLGAIERVVPREQLRDVAMEIANKIAAKSPA  200
ScdN            YFAHAHELHPRIAKEFLLLGERMPAERAYQMGMVNRVVPRAELQDQVYAMAQRMAAQPRL  218
                     : .    :  :.: *. : *  *   * :. : .   :   .   *: :*  .

ScdH            AVEQIKEVVIAGMDASLDAALMLERKANQILFATRDQKEGMNAFIEKRQPVFKGE-----  271
1DUB            IVAMAKESVNAAFEMTLTEGNKLEKKLFYSTFATDDRREGMSAFVEKRKANFKDH-----  261
ScdY            MIRIAKEALTGIEDGNLEDKYRWEQGFTLQAYMSPDSAETRSAFVEKRDAKF--------  252
ScdN            GLALTKMVVNKAEELQGLRSTMEMAFGYHHFAHAHSQAMGMGQLGGQDARSMAKANKEES  278
                 :   *  :    :                   : .     . :  :    :

ScdH            --
1DUB            --
ScdY            --
ScdN            KA                                                           280
```

Fig. S6 S6A: Expected position errors of $(ScdH)_6$ (S6A1), $(ScdY)_6$ (S6A2), and $(ScdN)_6$ (S6A3), and alignment of AlphaFold models of $(ScdH)_6$ and $(ScdY)_6$ (RMSD = 2.16 Å over 871 Cα atoms) (S6A4). In the expected position error plots, darker green indicates stronger predicted interactions between amino acid residues on the X- and Y-axes. S6A5: Clustal multiple amino acid sequence alignment of ScdH, enoyl-CoA hydratase from *Rattus norvegicus* (1DUB), ScdY, and ScdN. Amino acids highlighted in light blue boxes indicate substrate-binding regions suggested by the model shown in Fig. 6C2. Symbols: "*", identical; ":", highly similar; ".", similar.

S6B: Alignment and the pLDDT score of five top models

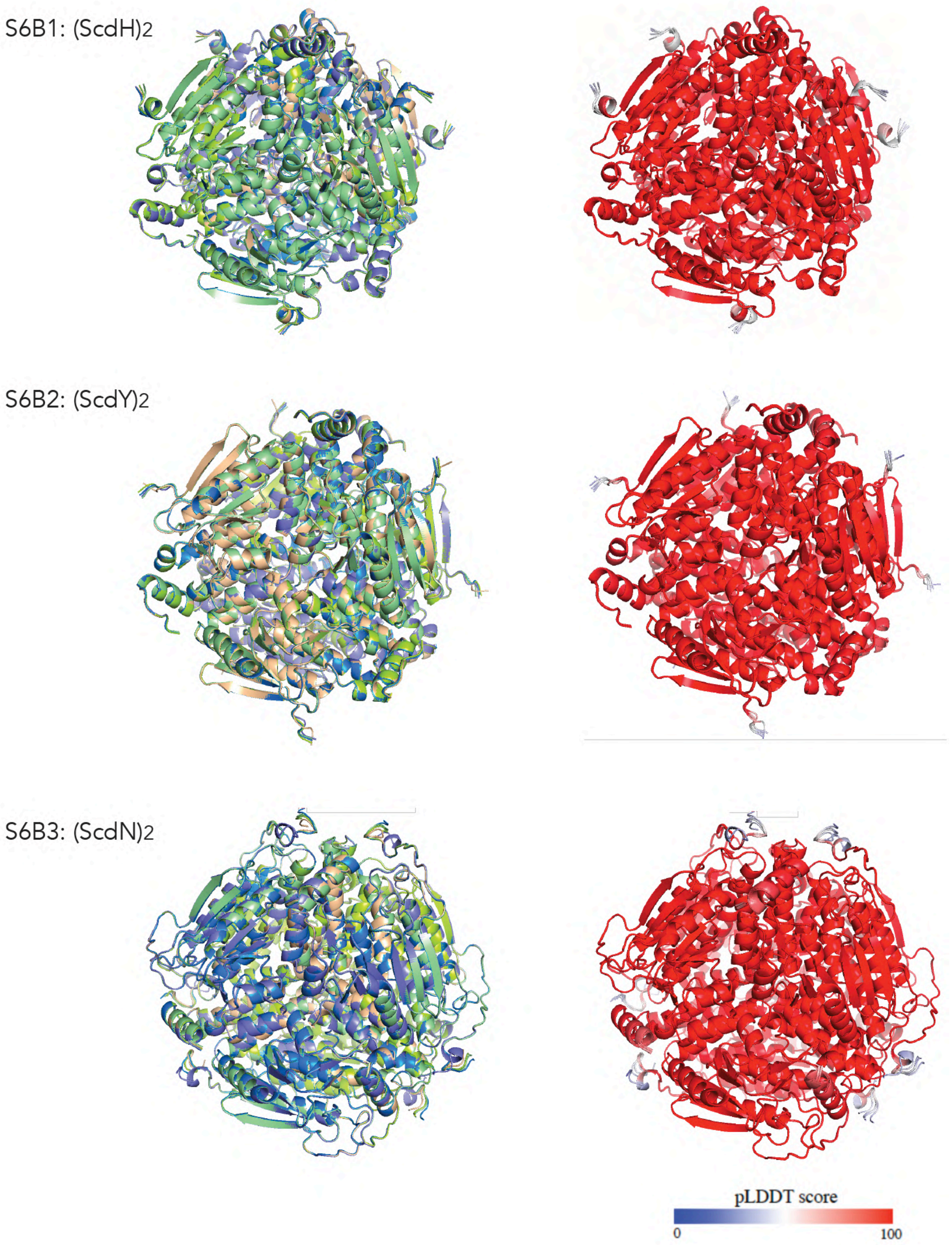

S6B: Alignment of the five top-ranked models and pLDDT scores of $(ScdH)_6$, $(ScdY)_6$, and $(ScdN)_6$ (S6B1–3). Color scheme: marine, model_0; slate, model_1; palegreen, model_2; limon, model_3; wheat, model_4.

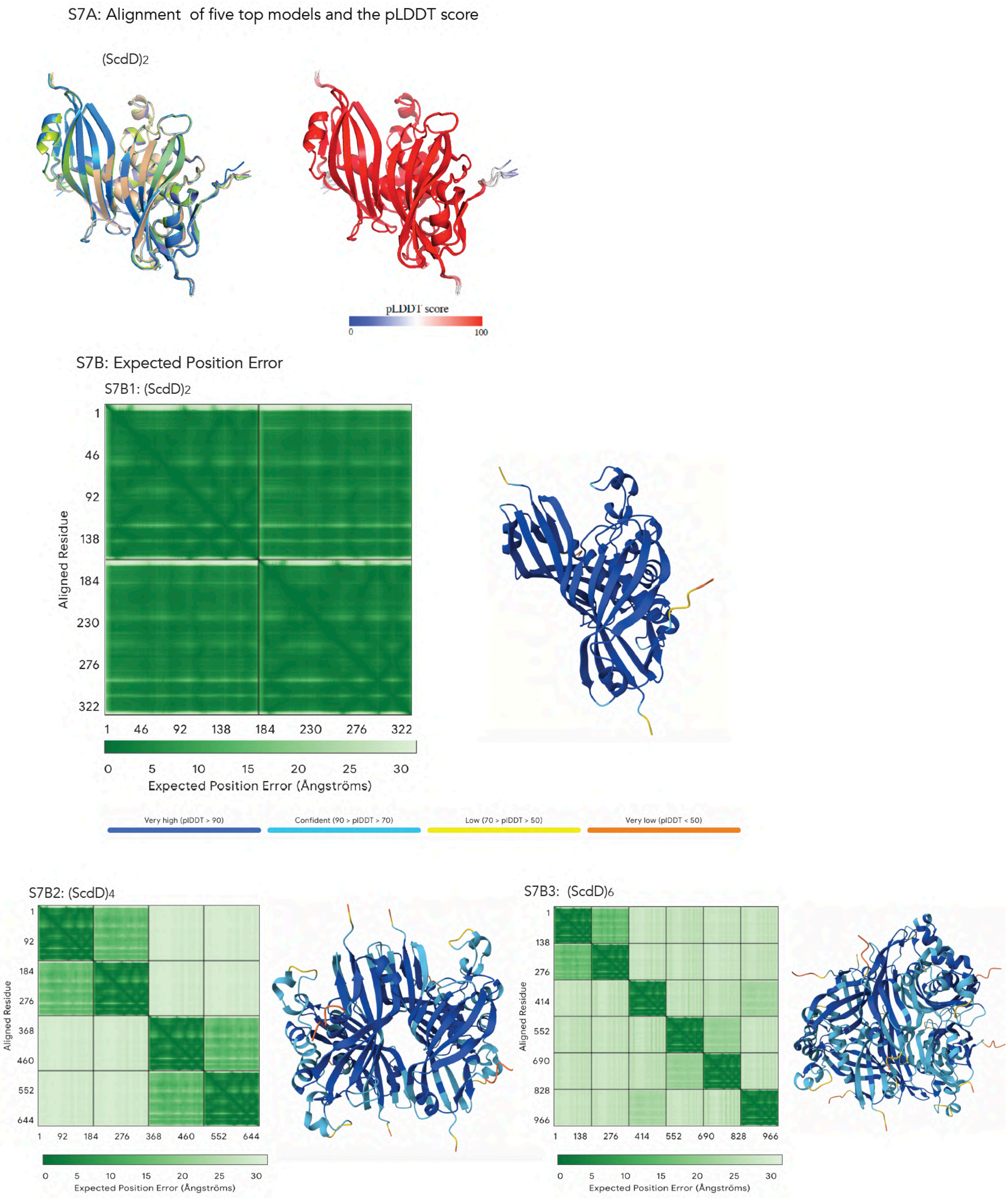

Fig. S7 **S7A:** Alignment of the five top-ranked models and pLDDT scores of $(ScdD)_2$. Color scheme: marine, model_0; slate, model_1; palegreen, model_2; limon, model_3; wheat, model_4. **S7B:** Expected position errors of $(ScdD)_2$ (S7B1), $(ScdD)_4$ (S7B2), and $(ScdD)_6$ (S7B3). In the expected position error plots, darker green indicates stronger predicted interactions between amino acid residues on the X- and Y-axes.

S7C1: CLUSTAL alignment of ScdD and ChsH2

```
ScdD     MNAVADKMLFDSAASVQASVGRQLGETEWVQITQERINQFADATGDHQWIHVDPEQARNG  60
ChsH2    -------MANQSIRFESVNVGDELQPVS-VPITVPLIAGGAIATRDYFPGHHDRDAAREL  53
                *  :*    ...** :*  .. * **   *   * ** *:   * * : **:

ScdD     PFGACVAHGYLTLSLANLFLPQLVSYQGLKMGVNYGCEKVRFPAPVLVNSFVRGSGEVVA  120
ChsH2    GS----PHIFMNILTTNGLVQSFVESWAGDDAR-LMDLKIRLGAPNYPGDTMKFTGSVTE  108
               .* ::.:  :* :: .:*.  . . .      *:*: **   .. :: :*.*.

ScdD     ATPIGEDGVQVTIRITVQVRNQDKPGCVVETISRLFFIREN
ChsH2    KN-------EATRSVQIALKGSNSMGSHVSGTVQVALA---
          .       :.*  : : ::..:. *. *.   :: :
```

S7C2: CLUSTAL alignment of ScdD and ChsH1

```
ScdD     -----MNAVADKMLFDSAASVQASVGRQLGETEWVQITQER-INQFADATGDHQWIHVDP  54
ChsH1    MAEVRAYVGKQYGRVYAWDKVNSPMIRQWSELMGVAVKTNKDGKTVAPPAMLQVWCMEGP  60
                .  :   . :  .*::.: ** .*   * :. ::  : .* .:  : *   .*

ScdD     EQARNGP----------FGACVAHGYLTLSLAN---------------------------  78
ChsH1    VQNNYPPGSTTENPYEVLKLIEAHDFPSTVAVNSELSFERDVLEGEDLYYTTRLEAISEE  120
          * .  *          :    **.: :   .*

ScdD     --------LFLPQLVSYQGLKMG---------------------------VNYGCEKVRF  102
ChsH1    KTTALGTGYFVTQIMEYFSIKAANGEDEKVGQLMFRVFKFRPANVQKAEPAQVAAPKIKR  180
                  *:.*::.* .:* .                           .: .. *::

ScdD     PAPVLVN---SFVRGSGE------------------------------------------  117
ChsH1    PAPGVSDDNRFFWEGLKEGKLLIQRCKSCGDLHHPPGPVCPKCHSFDWDAVEASGKGTVY  240
         *** : :    * .*  *

ScdD     ------------VVAATPIGEDGVQVTIRITVQVR----NQDKPGCVVETISRLFFIREN  161
ChsH1    SFVVMHYPEVPPFDHPNPIGLVELQEGVRLIAQLVGVKPGEVKIGQKVQVEFNTFDGDLT  300
                     .  ..***   :*  :*: .*:     .: * *  *:.  . *    .

ScdD     --------
ChsH1    LAQFRPVG
```

S7C: Clustal amino acid sequence alignment of ScdD and ChsH2 (S7C1) and ScdD and ChsH1 (S7C2). Amino acids highlighted in light blue boxes indicate substrate-binding regions suggested by the model shown in Fig. 6C2. Symbols: "*", identical; ":", highly similar; ".", similar.

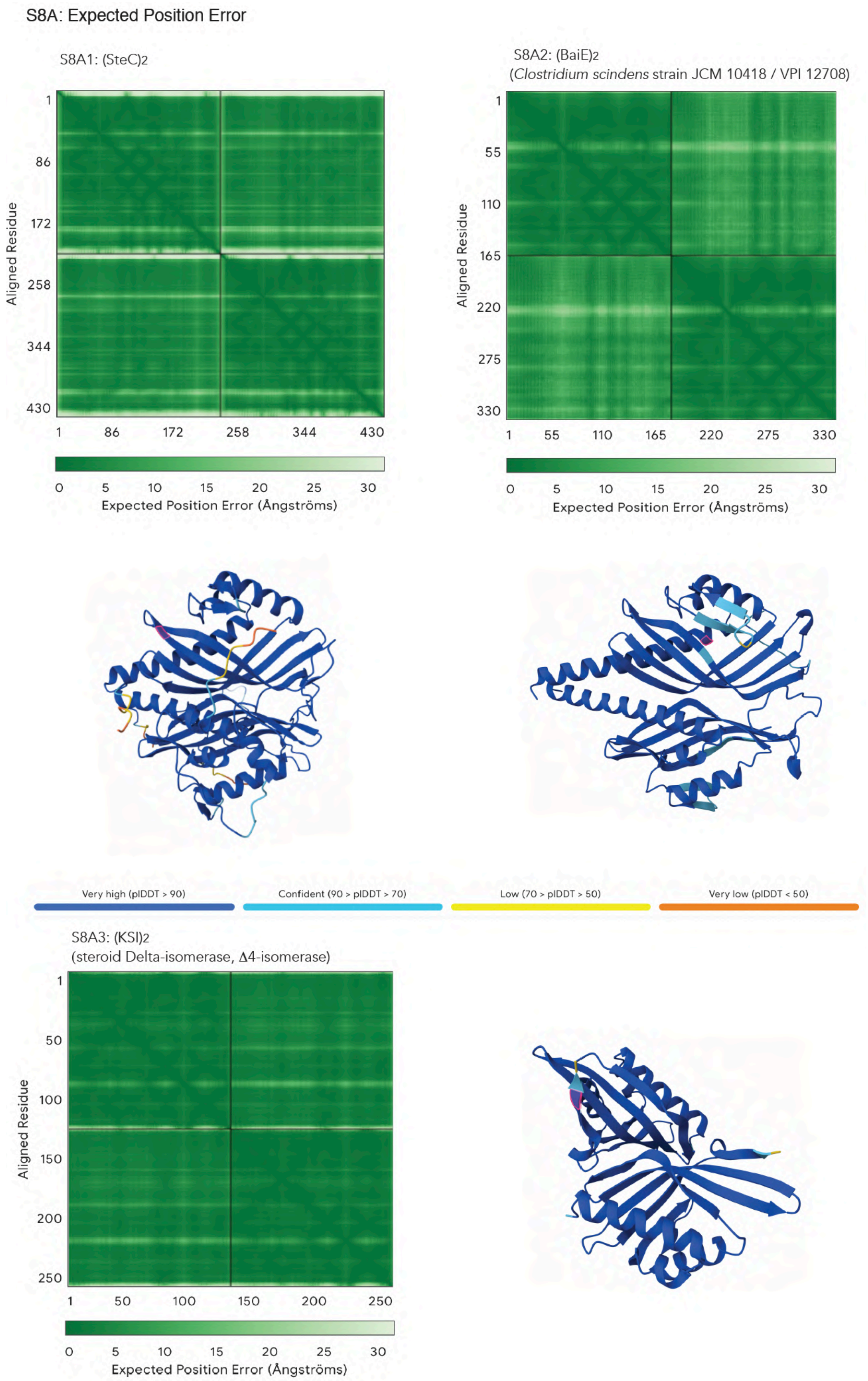

Fig. S8 **S8A:** Expected position errors of $(SteC)_2$ (S8A1), $(BaiE)_2$ (bile acid 7α-dehydratase from *Clostridium scindens* JCM 10418/VPI 12708, formerly *Eubacterium* sp. strain VPI 12708) (S8A2), and $(KSI)_2$ (ketosteroid Δ^4-isomerase) (S8A3).

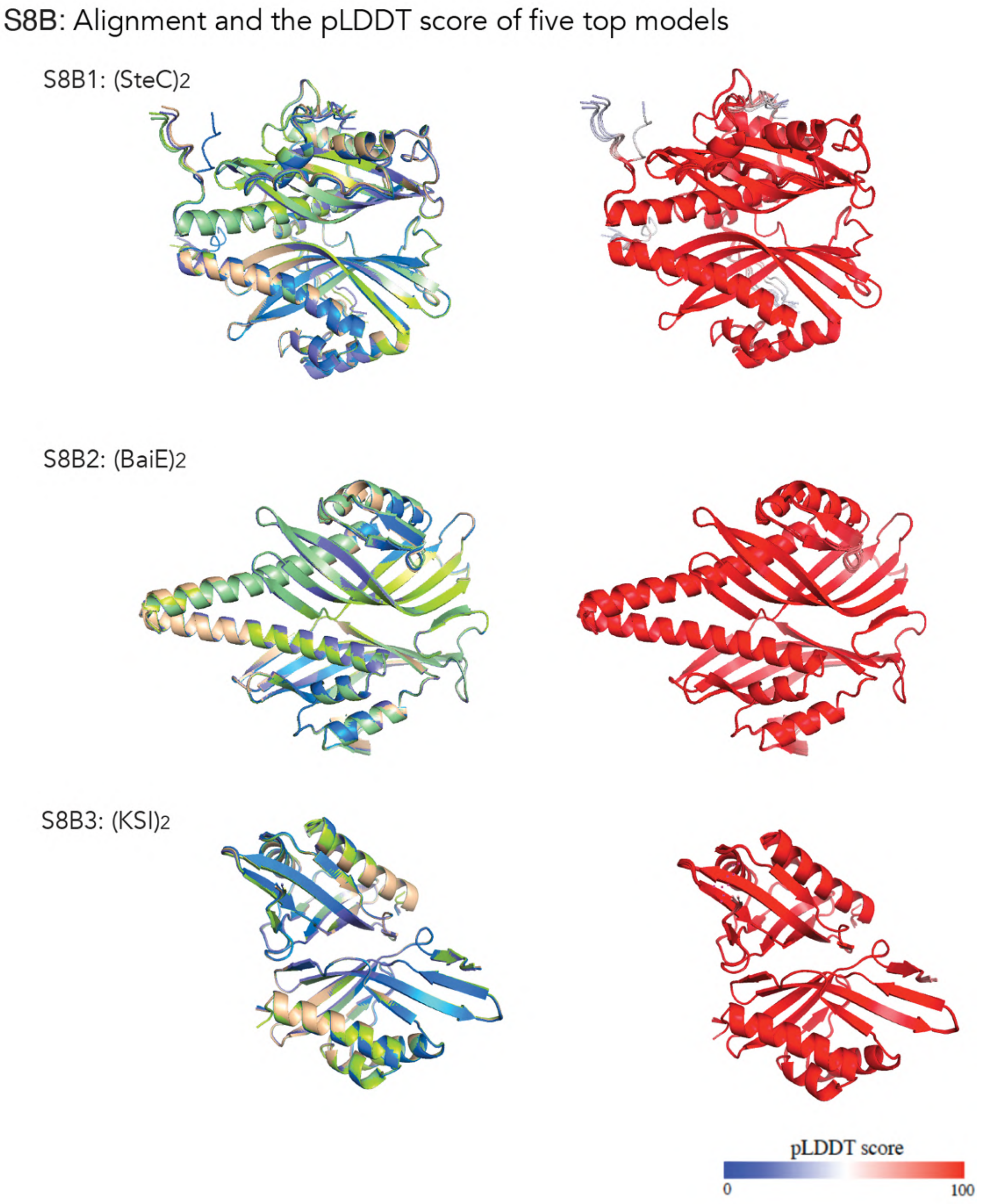

S8B: Alignment of the five top-ranked models and pLDDT scores of $(SteC)_2$ (S8B1), $(BaiE)_2$ (S8B2), and $(KSI)_2$ (S8B3).

S8C: CLUSTAL alignment of SteC and BaiE

```
SteC            MSEPVNQWPQTLEERIDRLESLDAIRQLAGKYSLSLDMRDMDAHVNLFAPDIKVGKEKVG   60
BaiE            --MGSDKIHHHHHHENLYFQGMTLEARIEALEKEIQRLNDIEAIKQLKAKYFRCLDGKLW   58
                     ::  :  ...   ::.:    :: .  .    :.*::*  :* *  ::  . *:

SteC            RAHFMAWQDSTLRDQFTG-TSHHLGQHIIEFVDRDHATGVVYSKNEHECGAEWVIMQMLY  119
BaiE            DELETTLSPNIETSYSDGKLVFHSPKEVTEYLAAAMPKEEISMHMGHTP--------EIT  110
                     : . .   .   *   .*  :.: *::    ..  :  :  *           :

SteC            WDDYERIDGQWYFRRRLPCYWYATDLNKPPIGDMKMRWPGREPYHGAFHDLFPSWKEFWA  179
BaiE            IDSENTATGRWYLED---------NLIFTDGKYKNVGINGGAFYTDKYEKIDGQWYIKET  162
                 *. :   *:**:.          :*  .     ::   *   * . :..:  .*    :

SteC            QRPDKDELPQVAAPAPLEQFLKSMRRGTPAPRMRVR  215
BaiE            GYVRIFEEHFMRDPKIHITSNMHKEK----------  187
                      *   :  *          .:
```

S8C: Clustal multiple amino acid sequence alignment of SteC and BaiE. Amino acids highlighted in light blue boxes form β-sheets and were used for alignment of the AlphaFold models of SteC and BaiE in Fig. 8C4. Symbols: "*", identical; ":", highly similar; ".", similar.

S8D: Expected Position Errors

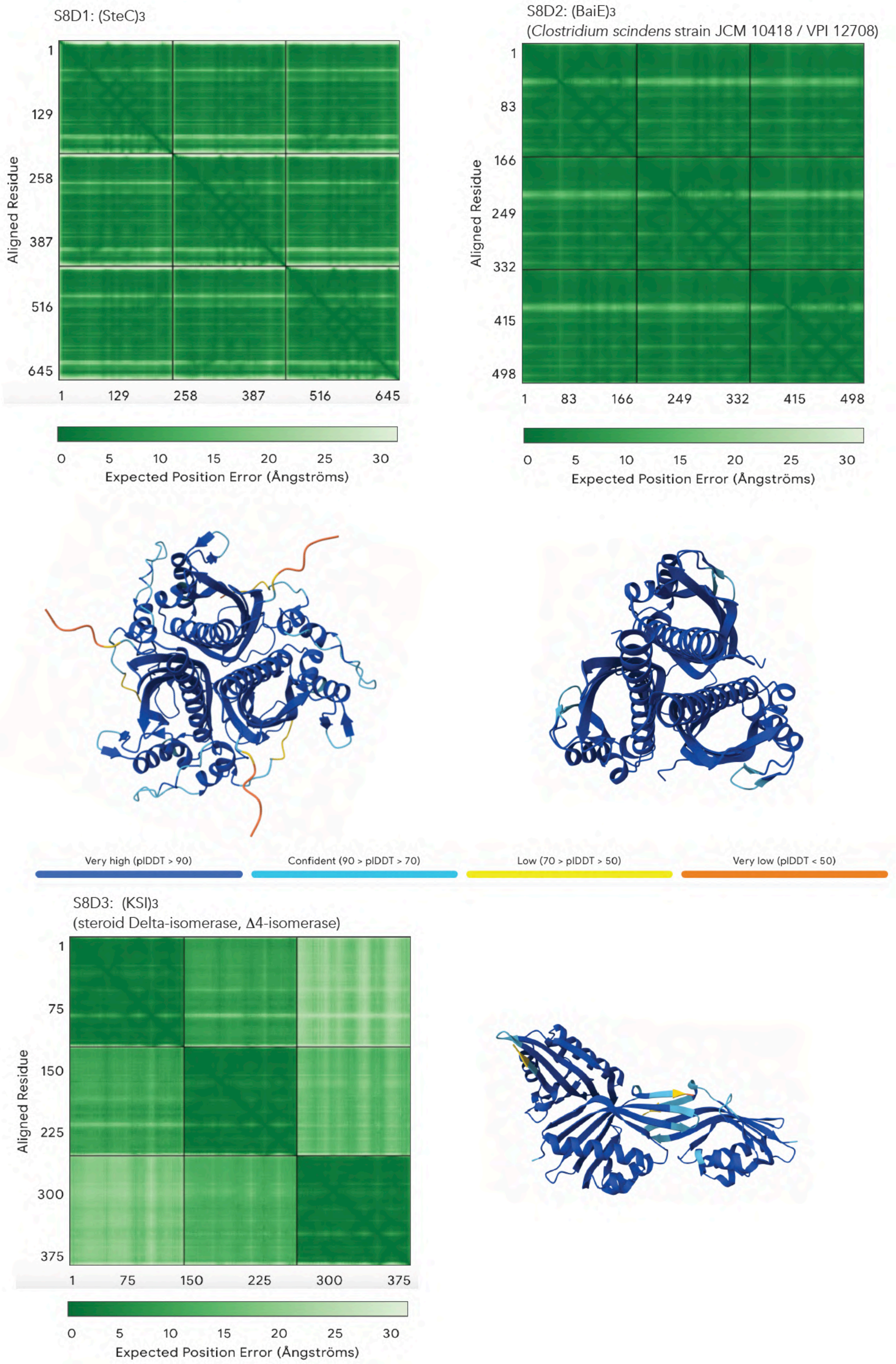

S8D: Expected position errors of $(SteC)_3$ (S8D1), $(BaiE)_3$ (S8D2), and $(KSI)_3$ (S8D3).

S8E: Expected Position Errors

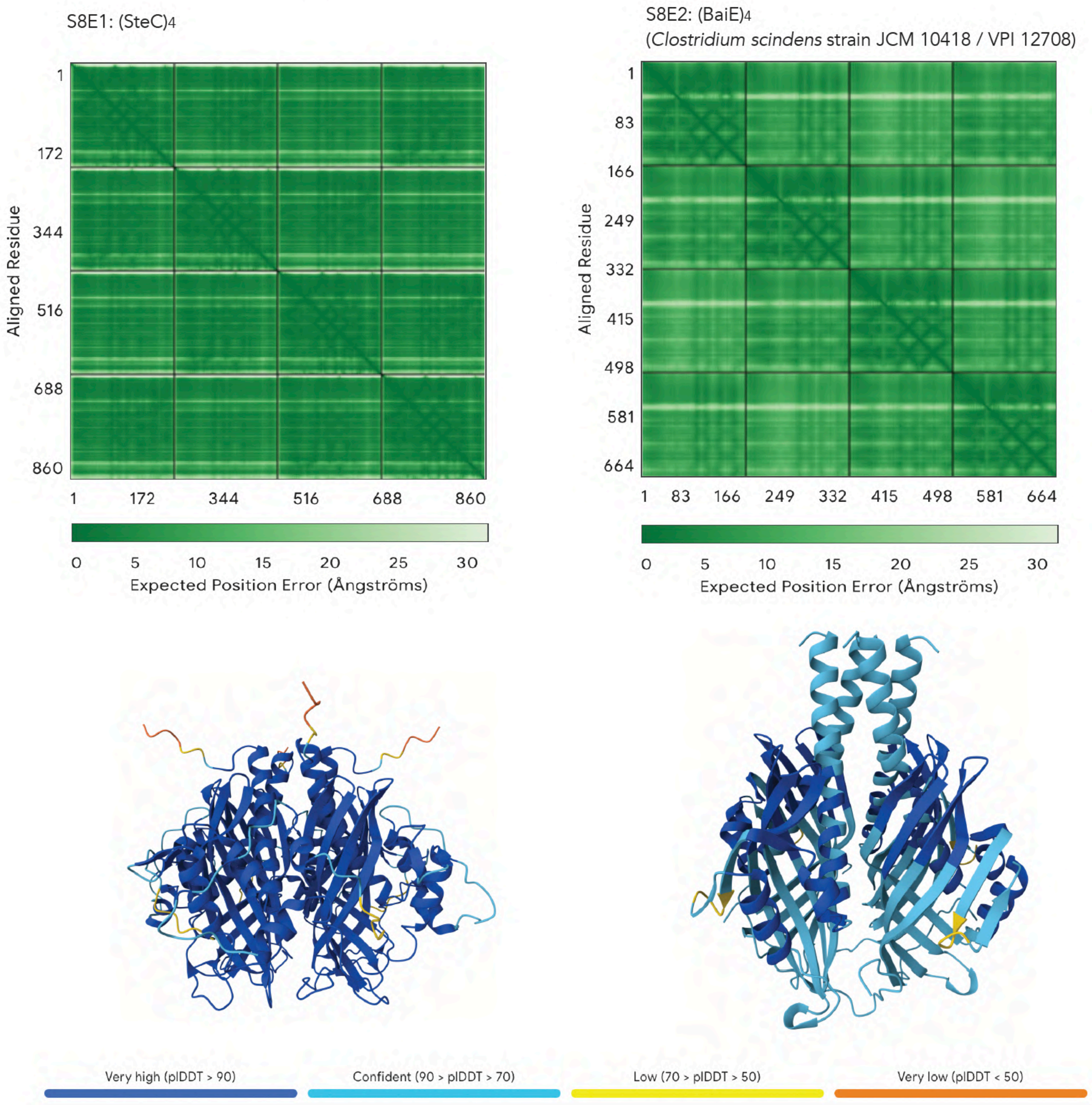

S8E: Expected position errors of $(SteC)_4$ (S8E1) and $(BaiE)_4$ (S8E2).

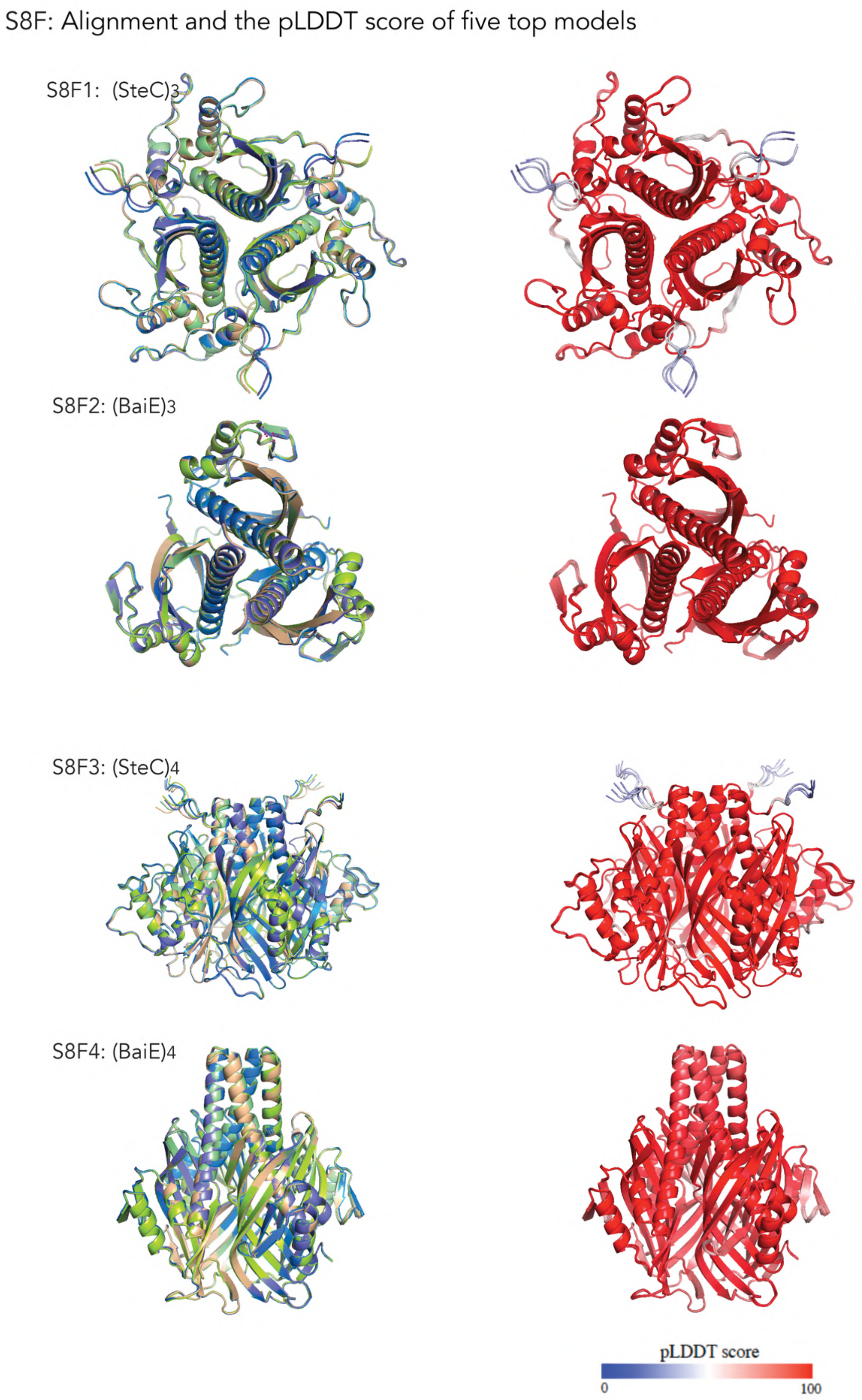

S8F: Alignment of the five top-ranked models and pLDDT scores of (SteC)$_3$ (S8F1), (BaiE)$_3$ (S8F2), (SteC)$_4$ (S8F3), and (BaiE)$_4$ (S8F4). Color scheme for the five-model alignments: marine, model_0; slate, model_1; palegreen, model_2; limon, model_3; wheat, model_4. In the expected position error plots, darker green indicates stronger predicted interactions between amino acid residues on the X- and Y-axes.

Table S1 plasmids

plasmids	Characteristics	Source or reference
pUC19	Ap^r, *lacZ*	(63)
pMFY42	Tc^r, Km^r, RSF1010-based broad host range plasmid	(45)
pMFYMhpRA	pMFY42 derivative carrying *mhpR* and the promoter of *mhp* genes	(26)
pMFYMhpORF38	pMFYMhpRA derivative carrying ORF38	this work
pMFYMhpORF39	pMFYMhpRA derivative carrying ORF39	this work
pUCORF38-Km^r	pUC19 derivative carrying DNA fragment containing ORF38: :Km^r	this work
pUCORF39-Km^r	pUC19 derivative carrying DNA fragment containing ORF39: :Km^r	this work
pUC37-42	pUC derivative carrying DNA fragment containing ORF37 to 42 (for construction of pUCORF39-Km^r)	this work
pUC35-39	pUC derivative carrying DNA fragment containing ORF35 to 39 (for construction of pUCORF38-Km^r)	this work

Km^r : Km-resistance
pSuperCosI* (Stratagene, CA)
(63) Vieira, J., and Messing, J. 1987. Methods Enzymol. **153:** 3-11.
(45) Nagata Y. et al. 1993. J Bacteriol **175**:6403-6410
(26) Horinouchi M, et al. 2023. Appl Environ Microbiol **89**:e0105023.

Table S2 primers

primers	Sequences	Source or reference
Dra_ORF38	TTTAAATGTCGGCATCGCTTTCCGC	this work
ORF38_Dra	TTTAAACTCCCCCTTGAACACCGGCT	this work
Dra_ORF39	TTTAAATGGCAGCAGCGCAGACTTC	this work
ORF39_Dra	TTTAAAGTGTTTTTGTTGCACGGATT	this work
ORF38_Km^r	CGGCGACAAGGT**GTTAAC***GGCAGGCGGCGA	this work
ORF38_Km^{r}RC	TCGCCGCCTGCC **GTTAAC***ACCTTGTCGCCG	this work
ORF39_Km^r	GCCACCGGCGTT**GTTAAC***GAGAACGTCCAG	this work
ORF39_Km^{r}RC	CTGGACGTTCTC **GTTAAC**sAACGCCGGTGGC	this work
MhpRPvuII_ORF38	GAGAATCTGGCCCAGATGTCGGCATCGCTTTCCGC	this work
MhpRPvuII_ORF39	GAGAATCTGGCCCAGATGGCAGCAGCGCAGACTTC	this work
ORF38_Km^{r}H	AGCCGGTGTTCAAGGATGATTGAACAAGAT	this work
ORF38_Km^{r}HR	ATCTTGTTCAATCATCCTTGAACACCGGCT	this work
ORF39_Km^{r}H	GTGCAACAAAAACACATGATTGAACAAGAT	this work
ORF39_Km^{r}HR	ATCTTGTTCAATCATGTGTTTTTGTTGCAC	this work
Km^r-MFYPvuII R	GGGGTGGGCGAAGAACTGGCAATTCCGGTT	this work

Km^r : Km-resistance
***GTTAAC**: *Hpa*I site
